# Opposing roles of CLK SR kinases in controlling HIV-1 gene expression and latency

**DOI:** 10.1186/s12977-022-00605-4

**Published:** 2022-08-19

**Authors:** Subha Dahal, Kiera Clayton, Terek Been, Raphaële Fernet-Brochu, Alonso Villasmil Ocando, Ahalya Balachandran, Mikaël Poirier, Rebecca Kaddis Maldonado, Lulzim Shkreta, Kayluz Frias Boligan, Furkan Guvenc, Fariha Rahman, Donald Branch, Brendan Bell, Benoit Chabot, Scott D. Gray-Owen, Leslie J. Parent, Alan Cochrane

**Affiliations:** 1 Dept. of Molecular Genetics, University of Toronto, 1 King’s College Circle, Toronto, ON M5S1A8 Canada; 2 Department of Pathology, University of Massachusetts Medical School, Worcester, MA 01605 USA; 3 Ragon Institute of MGH, MIT and Harvard, Cambridge, MA 02139 USA; 4 Dept. of Microbiology & Infectious Diseases, Université de Sherbrooke, Sherbrooke, QC Canada; 5 Department of Medicine, Penn State College of Medicine, Hershey, PA 17033 USA; 6 Microbiology & Immunology, Penn State College of Medicine, Hershey, PA 17033 USA; 7 Center for Innovation, Canadian Blood Services, Toronto, ON Canada

**Keywords:** HIV-1, RNA processing, SR kinases, Latency

## Abstract

**Background:**

The generation of over 69 spliced HIV-1 mRNAs from one primary transcript by alternative RNA splicing emphasizes the central role that RNA processing plays in HIV-1 replication. Control is mediated in part through the action of host SR proteins whose activity is regulated by multiple SR kinases (CLK1-4, SRPKs).

**Methods:**

Both shRNA depletion and small molecule inhibitors of host SR kinases were used in T cell lines and primary cells to evaluate the role of these factors in the regulation of HIV-1 gene expression. Effects on virus expression were assessed using western blotting, RT-qPCR, and immunofluorescence.

**Results:**

The studies demonstrate that SR kinases play distinct roles; depletion of CLK1 enhanced HIV-1 gene expression, reduction of CLK2 or SRPK1 suppressed it, whereas CLK3 depletion had a modest impact. The opposing effects of CLK1 vs. CLK2 depletion were due to action at distinct steps; reduction of CLK1 increased HIV-1 promoter activity while depletion of CLK2 affected steps after transcript initiation. Reduced CLK1 expression also enhanced the response to several latency reversing agents, in part, by increasing the frequency of responding cells, consistent with a role in regulating provirus latency. To determine whether small molecule modulation of SR kinase function could be used to control HIV-1 replication, we screened a GSK library of protein kinase inhibitors (PKIS) and identified several pyrazolo[1,5-b] pyridazine derivatives that suppress HIV-1 gene expression/replication with an EC_50_ ~ 50 nM. The compounds suppressed HIV-1 protein and viral RNA accumulation with minimal impact on cell viability, inhibiting CLK1 and CLK2 but not CLK3 function, thereby selectively altering the abundance of individual CLK and SR proteins in cells.

**Conclusions:**

These findings demonstrate the unique roles played by individual SR kinases in regulating HIV-1 gene expression, validating the targeting of these functions to either enhance latency reversal, essential for “Kick-and-Kill” strategies, or to silence HIV protein expression for “Block-and-Lock” strategies.

**Supplementary Information:**

The online version contains supplementary material available at 10.1186/s12977-022-00605-4.

## Plain Language Summary

Identifying cellular factors that regulate HIV-1 RNA processing provides important insights into novel strategies to control this infection. Different members of the SR kinase family have distinct roles in regulating virus expression because they affect distinct steps of transcription/RNA processing. We identify inhibitors of these kinases that suppress HIV-1 gene expression and replication in multiple assay systems at nanomolar concentrations with limited or no cytotoxicity. Our results highlight the therapeutic potential of targeting the post-integration stage of the HIV-1 lifecycle to selectively enhance or reverse provirus latency. A greater understanding of the molecular mechanisms underlying the effects observed will facilitate the development of more targeted approaches to modulate HIV-1 latency on the path toward a “functional” cure for this infection.

**Supplementary Information:**

The online version contains supplementary material available at 10.1186/s12977-022-00605-4.

## Introduction

HIV-1 gene expression is regulated at multiple levels (transcription, RNA processing, and RNA export to the cytoplasm) and critically depends on multiple host factors in addition to the viral encoded proteins Tat and Rev [1]. Following integration into the host cell genome, transcription of the provirus, mediated by several transcription factors and Tat, generates a single 9 kb pre-mRNA transcript. This full-length transcript undergoes alternative splicing to generate ~ 69 different viral mRNA species through usage of four splice donor sites and eight suboptimal splice acceptor sites [2–4]. The viral mRNAs produced are grouped as 9 kb unspliced (US) mRNA encoding Gag and Gag-pol; 4 kb singly spliced (SS) mRNA encoding Vif, Vpr, Vpu, Env, and Tat p14; and 2 kb multiply spliced (MS) mRNA encoding Tat p16, Rev, and Nef [1]. HIV-1 replication requires a precise balance of viral RNA processing to generate the proper proportion of genomic RNA for virions and viral mRNAs encoding viral structural proteins or viral factors to evade host innate defenses. Altering the balance has profound effects on viral gene expression and replication [2, 5–8]. HIV-1 RNA processing is orchestrated by an interplay between the *cis*-acting splicing regulatory elements (SREs) and the trans-acting cellular splicing factors that determine the frequency of individual splice site use [2, 5–8]. The major trans-acting cellular splicing factors that bind to the SREs are the serine-arginine rich proteins (SR proteins) and heterogeneous nuclear ribonucleoproteins (hnRNPs) [9–11].

SR proteins are a family of non-snRNP (small nuclear ribonuclear protein) splicing factors involved in both constitutive and alternative splicing [12–18]. They consist of one or two RNA recognition motifs (RRMs) at the N-terminus and a region rich in arginine and serine residues (RS domain) at the C-terminus [19, 20]. SR proteins use RRMs to interact with target RNAs, while the RS domain mediates protein–protein interaction to recruit components of core splicing apparatus and promote splice site pairing [13, 14]. Previous studies have reported roles for SR proteins in multiple stages of HIV-1 gene expression including regulation of viral transcription [21], Gag translation [22], virion production [23, 24], and modulation of HIV-1 RNA splicing (reviewed in [2, 5, 8, 25]). SR protein activity is primarily regulated by phosphorylation of serine residues in its RS domain that affect its protein–protein/protein–RNA interactions [16], intracellular localization/trafficking [26], and protein stability [27]. The phosphorylation status of SR proteins is also central to their effect on RNA metabolism including alternative splicing [28, 29]. Multiple kinases phosphorylate SR proteins, including SR protein-specific kinases (SRPKs), Cdc2 like kinases (CLKs), and dual-specificity tyrosine phosphorylation regulated kinases (DYRKs) [17, 19, 30, 31]. Comparison of SRPKs (SRPK1-2) and CLKs (CLK1-4) has revealed that, while they all phosphorylate residues within the RS domain, they differ in their specificity, extent of phosphorylation, protein sequences modified, and their subcellular localization [32, 33]. SRPKs are predominantly cytosolic and phosphorylate newly synthesized SR proteins to direct their transport to nuclear speckles which are storage sites for splicing factors [19]. Unlike SRPKs, CLKs are dual specificity kinases that phosphorylate serine and threonine, and autophosphorylate tyrosine residues [19, 29, 33, 34]. CLKs are extensively nuclear and regulate SR protein phosphorylation in the nucleus. Transient overexpression of CLKs in mammalian cells results in hyperphosphorylation of SR proteins, releasing them from nuclear speckles into the nucleoplasm [19, 33]. Previous studies have shown that small perturbations in the activity or abundance of key host factors critical for viral RNA processing can significantly impair virus replication [23, 35–37]. In particular, we previously showed differential effects of two CLK inhibitors, TG003 (CLK1 and 4 inhibitor) and chlorhexidine (inhibitor of CLK 2, 3 and 4), on HIV-1 gene expression [36, 38, 39]. Although TG003 had no effect, chlorhexidine effectively suppressed HIV-1 gene expression in micromolar doses in our cell-based assays, suggesting distinct roles of CLKs in modulating viral gene expression [36]. Multiple small molecule modulators of RNA processing, digoxin [37], chlorhexidine [36], 8-azaguanine [40], 5350150 [40], ABX464 [41], 1C8 [35], 9147791 [42], and 5342191 [43], are able to alter HIV-1 RNA accumulation and inhibit virus replication with very limited alterations to host RNA processing [35, 41–44]. Together, these observations highlight the delicate balance in viral RNA processing needed for HIV-1 replication and the capacity of various small molecules to disrupt this balance.

To expand our understanding of the role of different SR kinases in regulating HIV-1 gene expression, we examined the effects of depleting individual CLKs (1–3) and SRPK1 in different model cell lines and studied their impacts on HIV-1 gene expression and SR protein abundance. Here, we demonstrate unique roles of the individual SR kinase in the regulation of HIV-1 gene expression. Depleting CLK2 resulted in dramatic loss of viral gene expression while reducing CLK1 levels increased viral gene expression in two T cell lines containing HIV-1 integrated proviruses and enhanced the effect of various latency reversing agents (LRAs). While CLK3 depletion had limited effect, loss of SRPK1 also resulted in suppression of viral gene expression. Changes in SR kinase expression were also observed upon CD4+ T-cell activation, conditions known to elicit alterations in viral RNA processing associated with latency reversal. In parallel, we screened the GSK published kinase inhibitor set (PKIS) I and II and identified pyrazolo[1,5-b] pyridazine derivatives that modulate CLK1 and 2 function and effectively suppress HIV-1 gene expression at nanomolar doses. Together, our observations define the unique roles of the SR kinases in the regulation at specific steps of HIV-1 gene expression and demonstrate that selective modulation of SR kinase activity can be used to perturb HIV-1 gene expression to either block virus replication or reverse latency.

## Results

### Depletion of select SR kinases has differential effects on HIV-1 gene expression

**Fig. 1:**
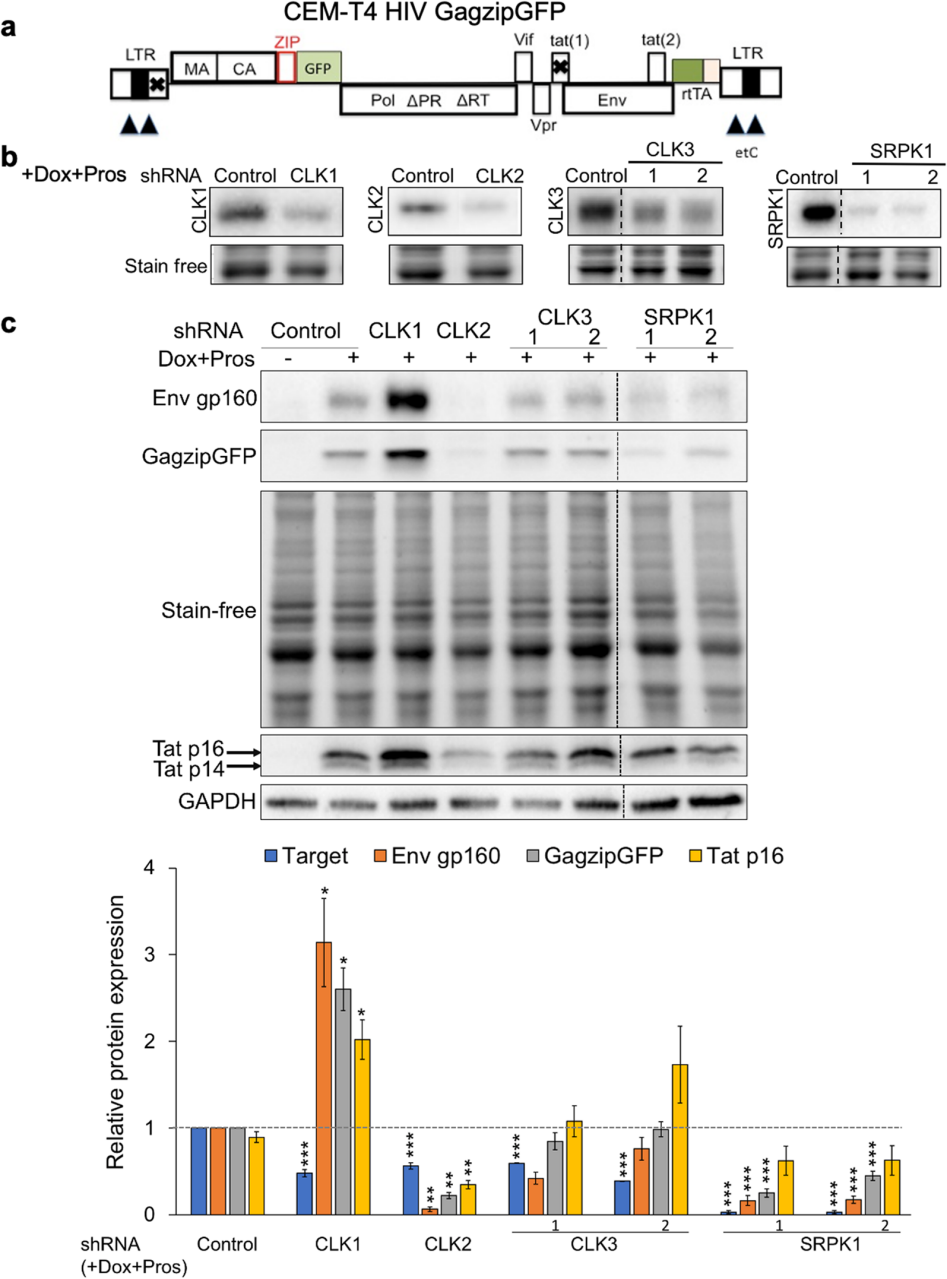
Depletion of SR kinases has differential effects on HIV-1 protein levels. **a** Schematic of HIV-1 rtTAGagzipGFP provirus used to generate CEM-HIV* cell line. **b**, **c** CEM-HIV* cells were infected with shRNA lentivirus targeting CLK1, CLK2, CLK3, or SRPK1 and transduced cells were selected with puromycin for 72 h. Following puromycin selection, HIV-1 gene expression was induced with doxycycline (Dox, 4.5 µM) + prostratin (Pros,2.56 µM) and cells harvested for western blots after 24 h of induction. Shown are the representative western blots indicating expression levels of **b** the target kinase, **c** or HIV-1 Env, Gag, and Tat levels. Band intensity was quantified relative to Dox induced shRNA control and normalized to either total protein stain for Env and Gag blots or GAPDH for Tat blots using Bio-Rad ImageLab software. Data are indicated as mean ± SEM, n ≥ 4 independent experiments, *p ≤ 0.05, **p ≤ 0.01, and ***p ≤ 0.001. Dotted vertical lines on the blots represent cropping of lanes on the same representative blot to show shcontrol lanes adjacent to shRNA target depletion lanes

To explore the role of select SR kinases in the regulation of HIV-1 gene expression, we individually depleted CLK1, CLK2, CLK3, or SRPK1 in the model cell line, CEM-T4 HIV Gag-zip-GFP (CEM-HIV*). This cell line harbors a modified HIV-1 provirus (Fig. 1a) in which the Nef reading frame has been replaced with reverse tetracycline-controlled transactivator (rtTA), tetracycline operator (tetO) sites inserted in the U3 region of the LTR [45, 46], and Tat function is inactivated by mutations within both the Tat reading frame and transactivation response (TAR) region (Fig. 1a). Together, these modifications render HIV-1 expression dependent upon addition of doxycycline (Dox) to the media. To examine the role of individual SR kinases in regulating HIV-1 expression, initial studies evaluated the capacity of five independent shRNAs to reduce target protein expression. Only those shRNAs that depleted target protein levels by > 50% of the control were used in subsequent assays. After confirming target protein depletion in cells (Fig. 1b), expression levels of the viral structural proteins Gag and Env, and regulatory protein, Tat, were determined via western blots (Fig. 1c). As expected, in the absence of Dox, cells did not express any viral proteins. In the presence of Dox, depletion of CLK3 expression by 40–60% had limited effects on all viral proteins probed. In contrast, a similar 40–60% reduction of CLK2 expression decreased viral protein levels; > 90% reduction in viral Env, ~ 80% reduction in Gag, and 65% reduction in Tat protein expression. Decreasing SRPK1 levels by more than 90% also resulted in reduced levels of both viral structural proteins, Env and Gag, but had limited to no effect on Tat (Fig. 1c). In contrast, depletion of CLK1 by ~ 50% in CEM-HIV* cells increased expression levels of all viral proteins; Env by threefold, Gag by 2.6-fold, and Tat by twofold. Parallel testing in the context of the J-Lat 10.6 cell line, containing a functional Tat/TAR transactivation circuit (Additional file 1: Fig. S1a), indicated similar responses upon depletion of the individual SR kinases to those seen in CEM-HIV* cells (Additional file 1: Fig. S1b). Together, these results reveal distinct effects of depleting select SR kinase on HIV-1 protein expression, with opposing roles of CLK1 and CLK2 in the control of viral protein expression in both cell lines examined.

### Altered HIV-1 protein expression upon SR kinase depletion correlates with changes in HIV-1 RNA accumulation

**Fig. 2:**
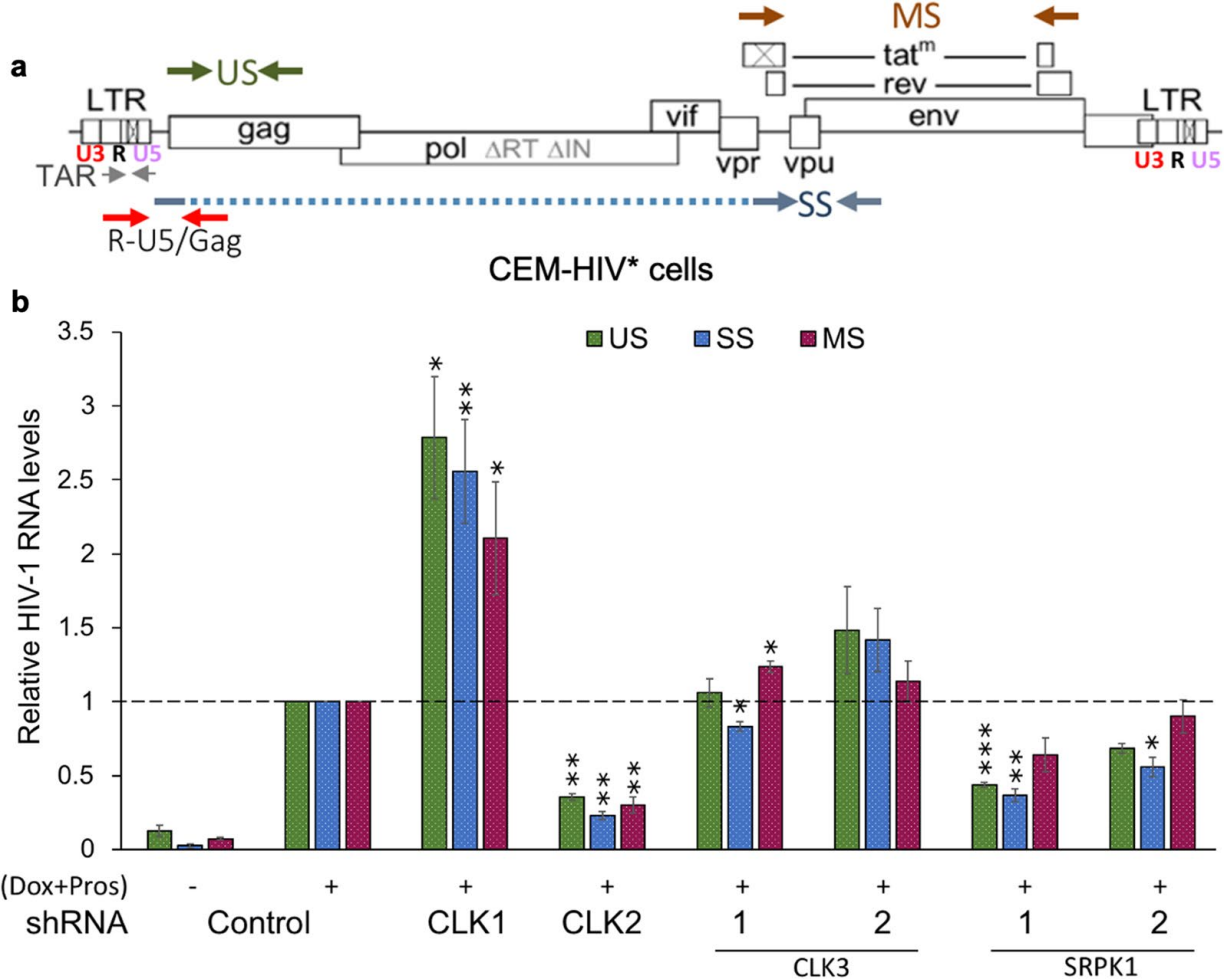
Effect of SR kinase depletion on HIV-1 RNA accumulation. **a** Schematic of HIV-1 provirus indicating the position of primers used to detect viral RNAs. **b** CEM-HIV* cells were depleted of CLK1, CLK2, CLK3, or SRPK1 by transduction with lentiviruses expressing shRNAs to these SR kinases. Following selection of transduced cells with puromycin for 72 h, HIV-1 gene expression was induced by addition of Dox (4.5 µM) + prostratin (2.56 µM). Cells were harvested for RNA extraction after 24 h of induction. HIV-1 unspliced (US), singly spliced (SS), multiply spliced (MS) RNA levels were determined by RT-qPCR. Viral mRNA levels were normalized to ß-actin and the mean mRNA levels expressed relative to sh control. Data are indicated as mean ± SEM, n = 3 independent experiments, *p ≤ 0.05, **p ≤ 0.01, and ***p ≤ 0.001

To determine whether the altered viral protein expression upon select SR kinase depletion correlates with changes in viral mRNA levels, the effect of depleting individual SR kinases on all three classes of HIV-1 RNAs was examined. Total RNA was extracted from cells depleted of individual SR kinases and RT-qPCR assays performed for HIV-1 US, SS, and MS RNAs in the CEM-HIV* cell line. The positions of the primers in the proviral construct are shown in Fig. 2a. CLK2 depletion reduced all classes of viral RNAs, US, SS, and MS RNAs, by more than 50% (Fig. 2b) consistent with the loss of Gag, Env, and Tat proteins, respectively (Fig. 1c). In contrast, depletion of CLK1 increased levels of all classes of viral RNAs (by more than twofold, Fig. 2b) consistent with the increased expression of viral Gag, Env, and Tat proteins observed (Fig. 1c). Depleting SRPK1 induced loss of both US and SS RNAs with limited effects on MS RNA levels (Fig. 2b) mirroring the effects observed in the western blots. CLK3 depletion had minimal effects on viral RNA levels consistent with the limited changes in viral protein expression observed (Fig. 2). To further examine if depletion of individual SR kinases alters the level of splice variants within HIV-1 MS RNA species, the HIV-1 MS RNA splicing pattern was analyzed by RT-PCR using forward and reverse primers that amplify the MS RNA spliced isoforms. No significant changes in splice site usage were observed with individual SR kinase depletion that would contribute to alterations in the viral Tat p16 expression observed (Additional file 1: Fig. S2a).

### Depletion of CLK1 but not CLK2/3 alters HIV-1 transcription initiation

**Fig. 3:**
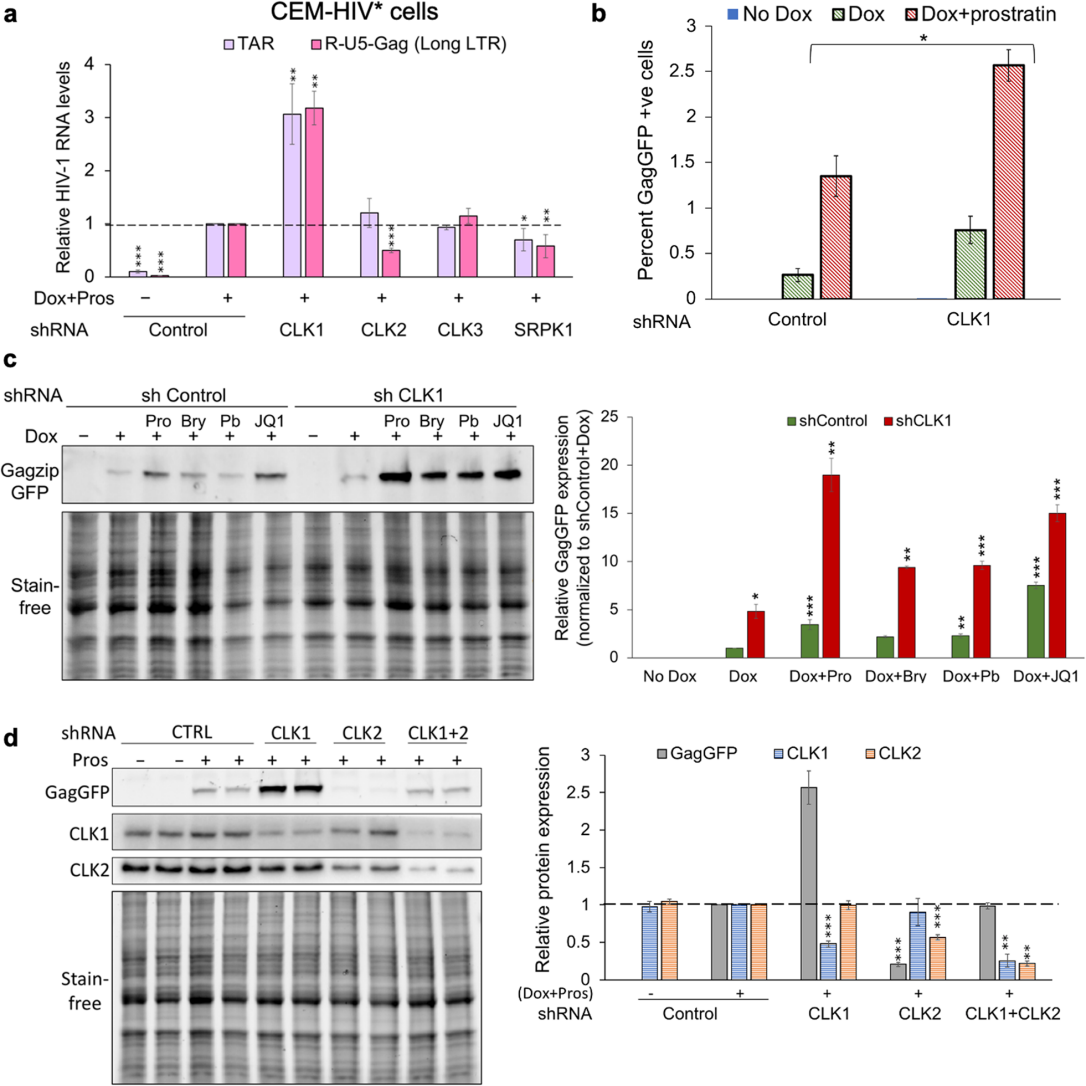
CLK1 but not CLK2/3 depletion alters HIV-1 transcription initiation and enhances response to LRAs. **a** Quantification of TAR and R-U5-Gag RNA levels in CEM-HIV* cells depleted of individual CLK1, 2, 3, or SRPK1 by shRNA lentivirus. Relative quantification was performed using comparative cycle threshold (CT) values. PUM1 was used as a reference gene to normalize the CT value and the fold changes calculated using 2^−ΔΔCT^ method. **b** CEM-HIV* cells were depleted of CLK1 by transduction with shRNA lentivirus and transduced cells selected with puromycin for 72 h. Cells were induced by addition of Dox only or both Dox + prostratin. Following induction of provirus expression, cells were fixed and the frequency of GagzipGFP positive cells determined by flow cytometry. **c** CLK1 depletion enhances the ability of different LRAs to promote HIV-1 protein expression. CEM-HIV* cells were depleted of CLK1 by transduction with lentiviruses expressing shRNA. Following selection of transduced cells with puromycin for 72 h, HIV-1 gene expression was induced by addition of Dox (4.5 µM) alone, or with Dox and an LRA-prostratin (Pro, 2.56 µM), bryostratin (Bry, 25 nM), panobinostat (Pb, 40 nM), or JQ1 (2 µM). Following induction for 24 h, cells were harvested, and cell lysates analyzed for effects on GagzipGFP expression. **d** Effect of CLK1/2 single and double depletions on GagzipGFP expression in CEM-HIV* cells. Infection with shRNA viruses, selection and provirus induction are as previously detailed. Representative western blots are shown on the left and a graphical summary of n > 3 assays on the right. Data are indicated as mean ± SEM, *p ≤ 0.05, **p ≤ 0.01, and ***p ≤ 0.001

Changes in HIV-1 RNA accumulation observed upon depletion of CLK1 or CLK2 could be achieved at multiple stages of viral RNA generation and processing; transcription initiation, elongation, splicing, transport or stability. Characterization of the CEM-HIV* cell system using digital RT-qPCR revealed that TAR RNA levels were almost 12-fold higher than that of R-U5-Gag RNA (Additional file 1: Fig. S3), consistent with known blocks in RNA polymerase II elongation upon initiation from the HIV-1 promoter [47]. A similar difference in TAR versus R-U5-Gag RNA levels has also been reported in both primary CD4^+^ T cells and J-Lat 10.6 systems and this difference has been interpreted to reflect the levels of initiation versus elongation from the HIV-1 promoter [48, 49]. To define the basis for the changes in viral gene expression observed upon depletion of individual SR kinases, we examined the effect of each manipulation on HIV-1 TAR and U5-Gag RNA levels (primer positions indicated in Fig. 2a), the former being a measure of initiation from the viral promoter and the latter, proximal transcript elongation [48, 50]. Despite the depletion of CLK2 resulting in dramatic changes to HIV-1 US, SS and MS RNA as well as viral protein accumulation, no significant change in TAR RNA abundance relative to the shRNA control was observed (Fig. 3a). However, CLK2 depletion reduced R-U5-Gag (long LTR) RNA abundance. In contrast, reduction of CLK1 expression increased both TAR and R-U5-Gag RNA abundance by ~ threefold (Fig. 3a). CLK3 depletion had no impact on TAR or R-U5-Gag RNA levels. Finally, SRPK1 depletion reduced TAR RNA levels by 30% and long LTR RNA(R5-gag) levels by 40% indicating that SRPK1 affected provirus transcription initiation as well as viral RNA accumulation (Fig. 3a).

To explore the basis for elevated HIV-1 expression upon CLK1 depletion in the context of the CEM-HIV* cell line, we also examined the frequency of HIV-1 expressing cells. In a cell population, elevated gene expression can arise by increasing expression in individual cells or the proportion of cells expressing the gene. Flow cytometry-based analysis of the CEM-HIV* cells revealed that only a small proportion of cells (< 1%) become GagGFP+ upon Dox addition, a proportion that could be increased by addition of the latency reversing agent (LRA), prostratin (Fig. 3b). Depletion of CLK1 increased the frequency of GagGFP+ cells upon addition of Dox alone (relative to control shRNA vector) that was further increased upon treatment with Dox and the PKC activator prostratin [51] (Fig. 3b).

To examine whether reduced CLK1 expression could affect the response to other LRAs, the ability of CLK1 depletion to enhance HIV-1 gene expression in CEM-HIV* cells upon addition of several classes of LRAs was tested. Addition of prostratin, panobinostat, or JQ1 alone increased GagGFP expression by 3, 2, and sevenfold, respectively, relative to Dox induced control (Fig. 3c). Depletion of CLK1 further enhanced the effect of LRAs on HIV-1 expression; prostratin, panobinostat, or JQ1 increasing GagGFP expression by 16, 10, and 14-fold, respectively, relative to Dox induced control (Fig. 3c).

To gain additional insight into the opposing roles of CLK1 and CLK2 in the control of HIV-1 expression, we also tested the effect of individual versus combined depletion of these kinases. As shown in Fig. 3d, while reduced expression of either CLK1 or CLK2 increased or decreased HIV-1 GagGFP expression, respectively, depletion of both proteins resulted in limited changes in GagGFP relative to control (Fig. 3d).

Given that CLKs are known to modify SR proteins and that SR proteins have roles in multiple stages of RNA synthesis and processing [5, 52], the differential effects of depleting individual SR kinases on HIV-1 gene expression might be explained by selective effects on SR protein abundance or activity. To examine this hypothesis, we assessed whether depletion of select SR kinases affects the abundance of individual SR proteins (SRSF1, 2, 3, 4, 5, 6, 7, 9, 10, and the SR-related protein Tra2ß), many of which have been implicated in regulating HIV-1 gene expression [5, 8]. In CEM-HIV* cells, depletion of CLK3 or SRPK1 expression had only modest effects on abundance of the SR proteins examined (Additional file 1: Fig. S4a). Despite the marked changes in HIV-1 RNA accumulation, CLK1 or CLK2 depletion altered abundance of only a few SR proteins; CLK1 depletion reducing SRSF9 by ~ 20% while CLK2 depletion increased SRSF4 by 1.5-fold and reduced SRSF6 levels by 25% (Additional file 1: Fig. S4a).

### Activation of primary CD4+ T cells alters the expression of CLK1 and SRPK1

**Fig. 4:**
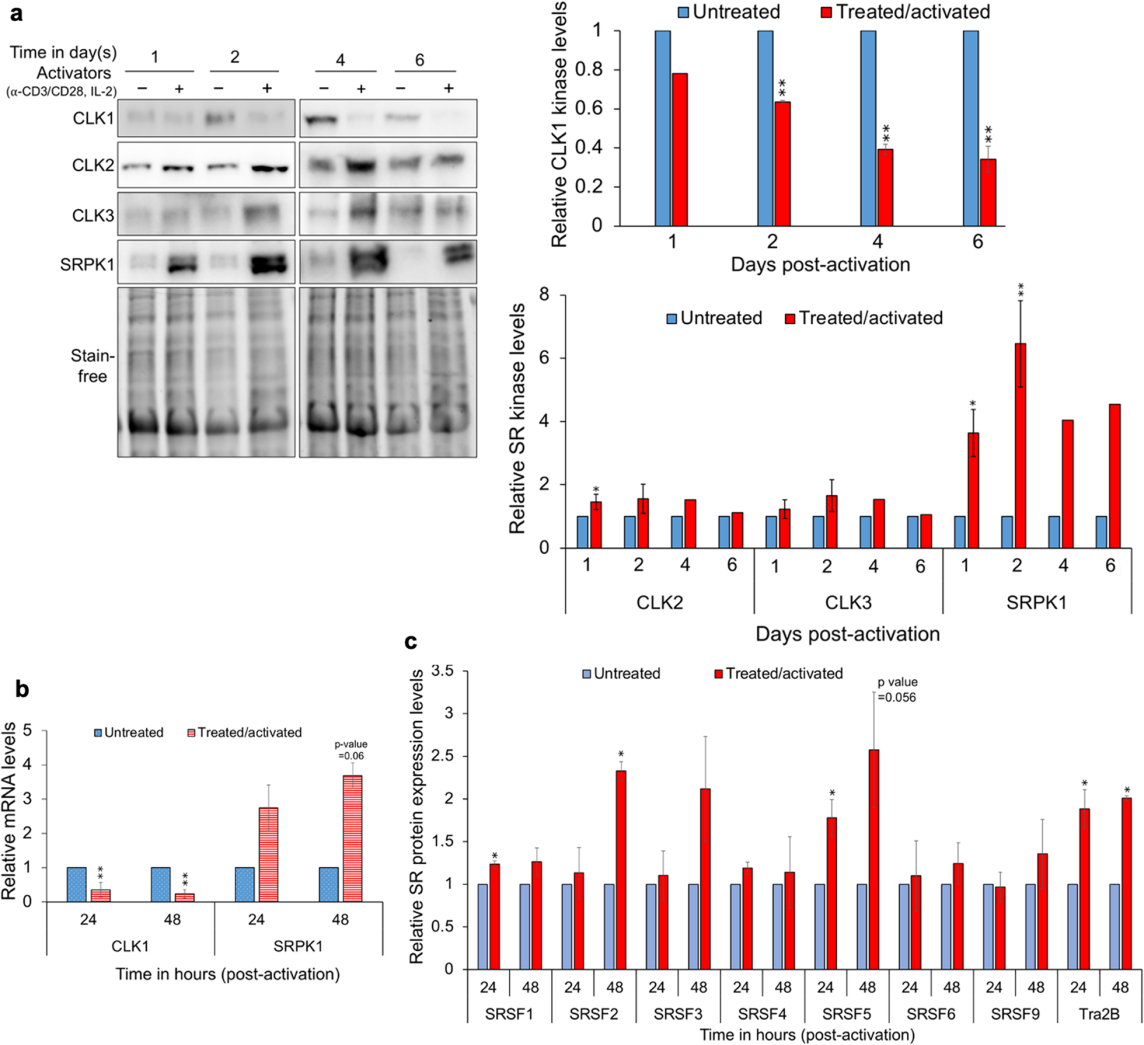
Activation of primary CD4+ T cells selectively alters SR kinase and SR protein levels. Primary CD4+ T cells were isolated from healthy (HIV uninfected) human donors and untreated (control) or treated with activators (anti-CD3/CD28 and IL-2). Cells were harvested at different times (24 h, 48 h, 4 d, and 6 d) with or without activation for analyses by western blots or RT-qPCR to look for changes in the expression of SR kinases and SR proteins. **a** Top and bottom panels on the left are the representative western blots probed for CLK1, CLK2, CLK3, and SRPK1. Top and bottom panels on the right are the quantitation of blots for at least 3 donors (except for 4 d and 6 d post-activation for CLK2, CLK3, and SRPK1 expression levels where only one donor was used). **b** Quantification of CLK1 and SRPK1 mRNA levels in CD4+ T cells of 3 donors by RT-qPCR assay. mRNA levels were normalized to ß2-microglobulin and mean mRNA levels were expressed relative to untreated control. **c** Quantitation of western blots for SR protein expression levels in untreated versus treated/activated CD4+ T cell lysates (see Additional file 1: Fig. S4 for representative western blots) across at least 3 donors. For western blots, band intensity was quantified relative to untreated control and normalized to total protein load using Bio-Rad ImageLab software. Data are indicated as mean ± SD, n = 3 or 4 independent experiments, *p ≤ 0.05 and **p ≤ 0.01

In light of the opposing roles of CLK1 and CLK2/SRPK1 in modulating HIV-1 gene expression, we looked for conditions which perturbed their relative expression in the context of primary CD4^+^ T cells. Previous studies have established that one of the most potent means of reversing HIV-1 provirus latency is through activation of T-cell receptor signaling mimicked by treatment with anti-CD3/CD28 antibodies and IL-2 [48]. This treatment induces marked changes in HIV-1 transcript elongation and RNA processing with more limited effects on transcription initiation [48]. To determine whether this treatment also elicited changes in SR kinase expression, the effect of anti-CD3/CD28 activation of primary CD4^+^ T cells on CLK1-3 and SRPK1 levels was examined. As shown in Fig. 4a, activation of CD4^+^ T cells resulted in limited or no alteration in CLK2 and CLK3 expression levels up to 6 days post activation. In contrast, activation resulted in a fourfold increase in SRPK1 protein levels within 1 day (peaking at sevenfold 2 days post activation) and a gradual decrease in CLK1 expression levels, reaching 60% of untreated levels 2 days post-activation then to ~ 40% of the untreated cells 4–6 days post-activation (Fig. 4a). To decipher the basis for the changes in CLK1 and SRPK1 expression, RNA samples from cells with or without activation were analyzed by RT-qPCR 24 h (1 day) and 48 h (2 days) post-activation. Consistent with the changes in CLK1 protein levels, abundance of CLK1 mRNA levels decreased 24 h post-activation and further declined to 20% of unstimulated cells 48 h post-activation (Fig. 4b). In contrast, SRPK1 mRNA levels increased by ~ 3- and ~ 4-fold at 24 and 48 h post-activation, respectively (Fig. 4b). T cell activation also resulted in selective changes in SR protein abundance (Figs. 4c, Additional file 1: Fig. S5); SRSF5 and Tra2ß levels increasing 24 h (1 day) after stimulation while SRSF2 and SRSF3 abundance increased only 48 h (2 days) post stimulation. The changes in SRPK1 and CLK1 expression, the factors that have opposing roles in regulating HIV-1 gene expression, likely contribute to generating a cell state more supportive of HIV-1 replication.

### Identification of potent inhibitors of HIV-1 Gag expression

**Fig. 5:**
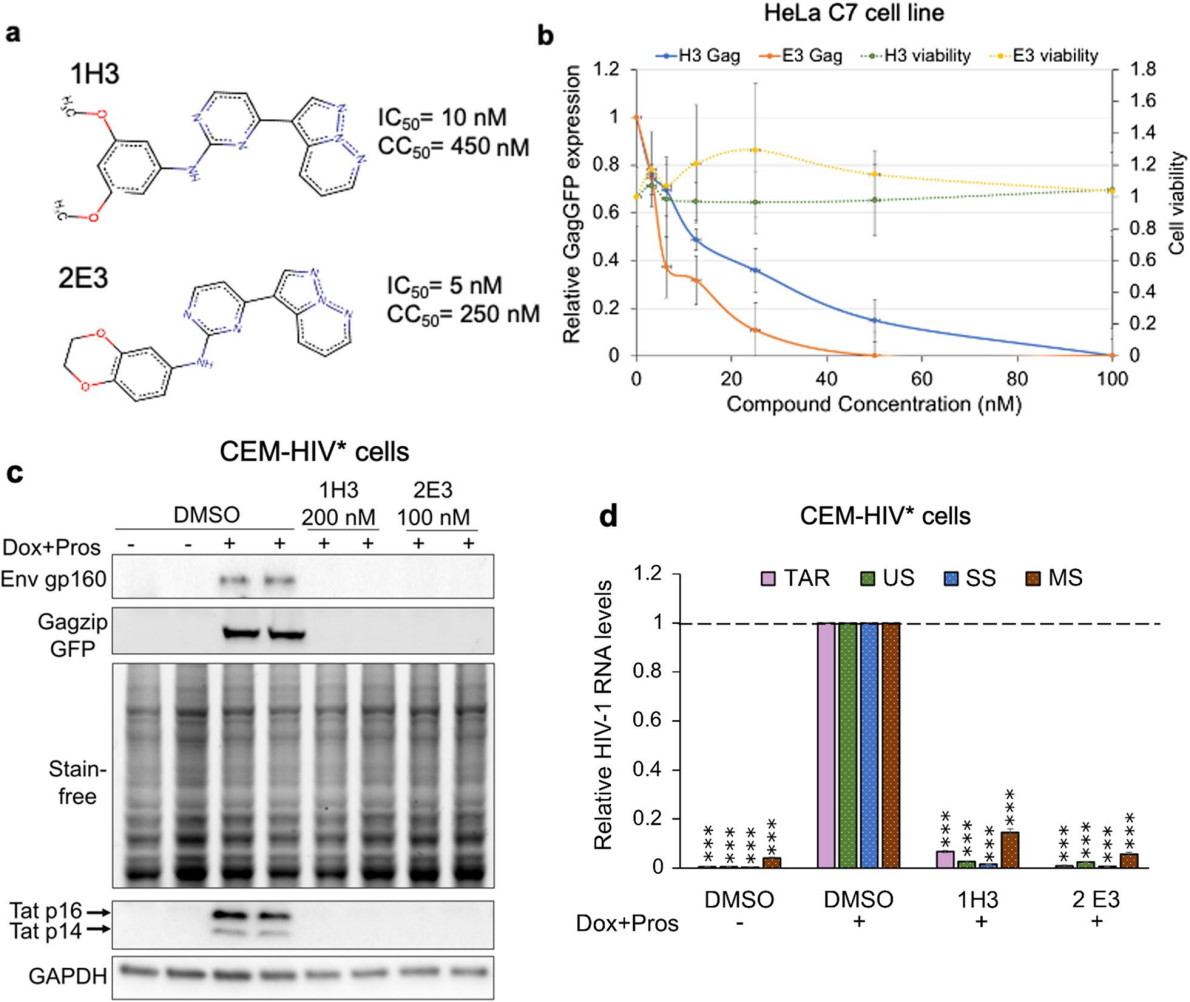
Identification of inhibitors of HIV-1 Gag expression from the GSK PKIS library. **a** Structures of 1H3 and 2E3. Indicated are the EC_50_ and CC_50_ values of the compounds as determined from assays using HeLa C7 cells; **b** HeLa C7 cells were incubated with compounds at increasing compound concentration and HIV-1 gene expression induced with Dox (4.5 µM) for 24 h. 1% DMSO treated cells grown with or without Dox served as positive and negative controls, respectively. Dose response on HIV-1 gene expression was measured relative to intracellular GagGFP levels in DMSO & Dox-treated samples. Effects of compounds on cell viability were assessed using alamarBlue assay across n > 3 independent assays. **c**, **d** CEM-HIV* cells were treated with compounds 1H3 (200 nM) or 2E3 (100 nM) and HIV-1 gene expression induced with Dox and prostratin. 1% DMSO treated cells grown with or without Dox and prostratin served as positive and negative controls, respectively. Cells were harvested for HIV-1 protein and RNA analyses after 24 h of induction. **c** Shown are representative western blots showing the effect of compounds on HIV-1 Gag, Env, and Tat levels. **d** Quantification of viral TAR, US, SS, and MS RNA levels in cells treated with compounds relative to induced DMSO control. The positions of the primers in the proviral construct are shown in Fig. 2A. Viral mRNA levels were normalized to PUM1 for TAR RNA and ß-actin for US, SS, and MS RNA. Mean mRNA levels were expressed relative to Dox and Prostratin induced DMSO control. Data are indicated as mean ± SEM, n = 4 independent experiments, *p ≤ 0.05, **p ≤ 0.01, and ***p ≤ 0.001

Our previous studies identified chlorhexidine, an inhibitor of CLK2, 3, and 4, as a suppressor of HIV-1 replication through its effects on viral RNA accumulation [36, 39]. This evidence, coupled with our demonstration that depletion of select SR kinases alter HIV-1 gene expression, indicates that inhibition or enhancement of HIV-1 replication might be achieved by using small molecule inhibitors of a subset of these kinases. To identify additional kinase inhibitors able to suppress HIV-1 gene expression, we screened the GSK published kinase inhibitor set (PKIS) I and II [53] in the HeLa rtTA HIVGagGFP (HeLa C7) cell line [54, 55] which contains an integrated provirus expressing a GagGFP fusion protein. To validate and identify the most active compounds, hits from the initial screen were retested and the two most potent compounds identified were pyrazolo[1,5-b] pyridazine derivatives, designated as 1H3 (GW801372X) and 2E3 (GW806290X) (Fig. 5a). Subsequent analysis of the dose response curves for these compounds and derivatives thereof demonstrated potent inhibition of HIV-1 Gag expression with an EC_50_ < 20 nM and minimal toxicity up to 200 nM (Fig. 5b, Additional file 1: Table S1).

To further evaluate the activity of 1H3 and 2E3, we examined their effects on HIV-1 protein levels and viral RNA accumulation. In the CEM-HIV* cell line, both compounds inhibited HIV-1 Gag, Env, and Tat levels to undetectable levels compared to the DMSO control at nanomolar doses (1H3 at 200 nM, 2E3 at 100 nM) (Fig. 5c). Loss of viral protein expression correlated with a reduction in abundance of all HIV-1 RNAs (TAR, US, SS, and MS RNAs, Fig. 5d). Subsequent examination of HIV-1 MS RNA by RT-PCR revealed no significant change in splice site usage upon 1H3 or 2E3 treatment relative to DMSO (Additional file 1: Fig. S2b). Additional testing of the compounds in the context of J-Lat 10.6, a Jurkat based T-cell line harboring a latent HIV-1 provirus that expresses GFP from the Nef position [56], revealed a similar reduction in viral gene expression (Additional file 1: Fig. S1c, d). Both 1H3 and 2E3, at the doses of 200 and 100 nM, respectively, reduced HIV-1 Gag and GFP expression to near undetectable levels and reduced accumulation of US and SS RNAs with some accumulation of MS RNAs. Together, these findings demonstrate that compounds 1H3 and 2E3 are potent inhibitors of HIV-1 gene expression, affecting both early (Tat, Nef) as well as late (Gag, Env) viral gene expression.

### 1H3 inhibits HIV-1 protein expression and replication in primary cells

**Fig. 6:**
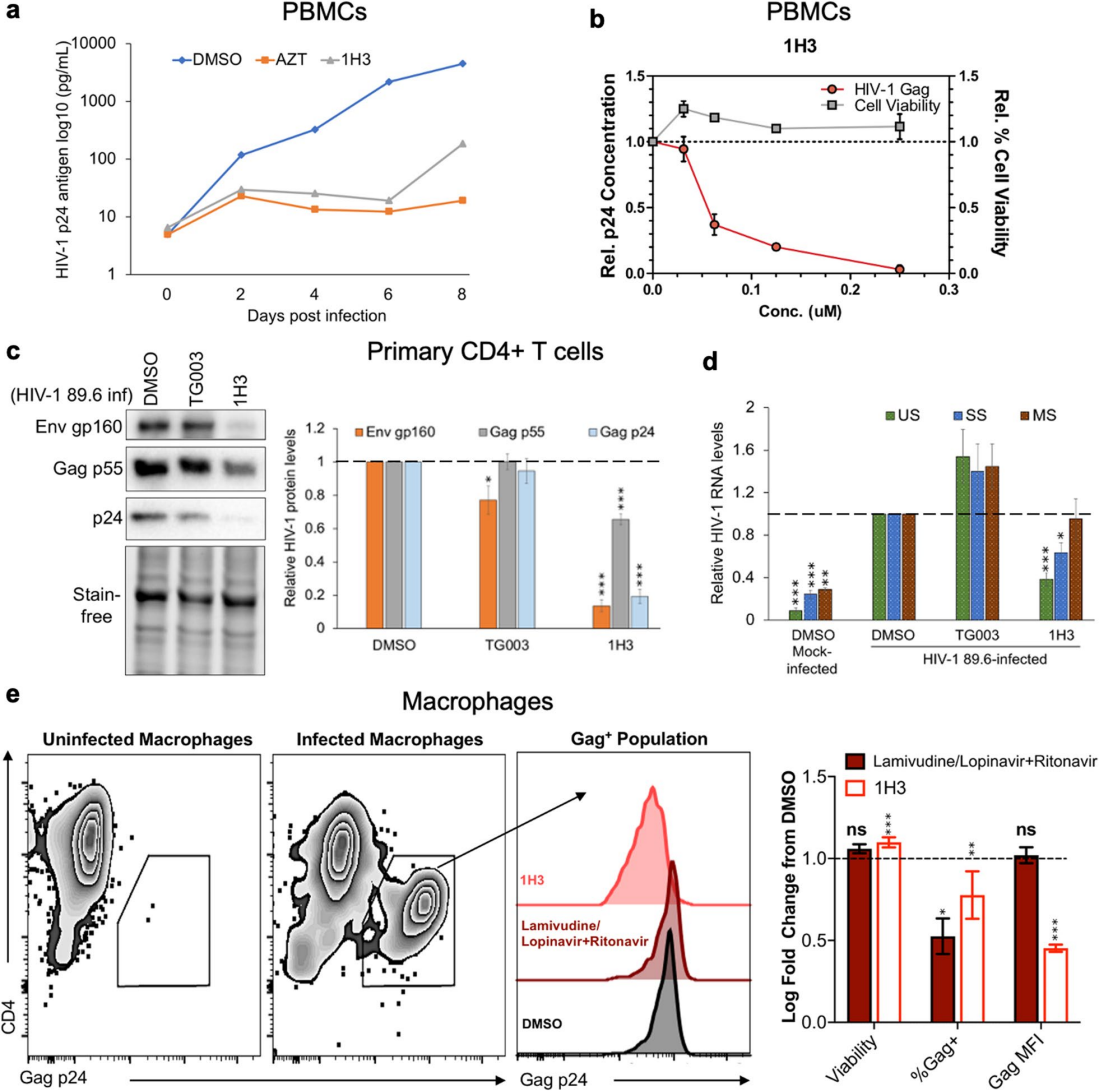
Compound 1H3 inhibits HIV-1 replication in PBMCs, CD4+ T cells, and macrophages. **a** HIV-1 BaL replication in PBMCs over a period of 8 days post infection as measured by p24 antigen ELISA. PBMCs from healthy human donor were infected with HIV-1 BaL (MOI < 0.01) and treated on day 0 and day 4 post infection with DMSO, AZT (3.74 µM), or 1H3 (0.25 µM). **b** The effect of increasing concentration of 1H3 on HIV-1 BaL virion production in PBMCs. Indicated doses of the compounds were added following infection, media harvested after 6 days, and virus replication measured by p24 ELISA. Values are expressed relative to p24 levels in DMSO-treated cultures at day 6. The effect of the compounds on cell viability was measured by trypan blue exclusion assays using Glasstic slides (Kova). n = 3. **c**, **d** CD4+ T cells from healthy human donors were infected with HIV-1 89.6 then treated immediately with DMSO, 10 µM TG003, or 300 nM 1H3 for 3 days. Cells were harvested and the effect of individual treatments on HIV-1 **c** protein or **d** RNA accumulation assessed by western blot and RT-qPCR, respectively. Shown are results from 6 independent experiments with 8–10 donors. **e** Effect of 1H3 on HIV-1 replication in human macrophages. Monocyte-derived macrophages from healthy donors were infected for 2 days with HIV-1 89.6, then treated with DMSO, a combined antiretroviral drug Lamivudine/Lopinavir + Ritonavir (1.5 µM/53 nM) or 1H3 (300 nM). After 10 days, cells were fixed, stained to detect intracellular levels of Gag and analyzed by flow cytometry to assess changes in %Gag positive cells and Gag mean fluorescence intensity (MFI). Shown on the left are representative flow cytometry plots of uninfected and infected macrophages and histograms showing shifts in the Gag Mean fluorescence Intensity (MFI) with treatment. On the right, summary data shown are expressed relative to DMSO treated samples (n = 8 from 5 independent experiments). Statistical analysis, one sample t test, ns = not significant, *p < 0.05, **p < 0.01, and ****p < 0.0001

To further validate the anti-HIV-1 activity of 1H3, its ability to inhibit replication of an R5 strain of HIV-1 (BaL) in peripheral blood mononuclear cells (PBMCs) was examined. PBMCs obtained from healthy donors were infected with HIV-1 BaL at a multiplicity of infection of 10^–2^, as previously described [42], and treated with either DMSO, azidothymidine (AZT), or 1H3. Following infection, virus replication was monitored by p24 antigen ELISA over a period of 8 days. Addition of 1H3 dramatically suppressed HIV-1 replication, to a level comparable to that seen with AZT (Fig. 6a). Parallel examination of the dose response curve of 1H3 demonstrated inhibition of HIV-1 replication with an EC_50_ of ~ 50 nM with no effects on cell viability (Fig. 6b) as measured by Trypan blue exclusion assays at concentrations up to 250 nM. Consistent with these findings, treatment of HIV-1_89.6_-infected CD4^+^ primary T cells (an X4R5 macrophage-tropic primary strain of HIV-1) with 1H3 reduced expression of HIV-1 Env and Gag proteins as well as decreased accumulation of viral US and SS RNAs (Fig. 6c, d). In contrast, TG003, another CLK inhibitor [38], had little or no effect on HIV-1 protein expression or RNA accumulation. Subsequent assays in primary human monocyte-derived macrophages infected with HIV-1_89.6_ took advantage of the ability of macrophages to survive the cytopathic effects of infection [57–61], allowing for the assessment of long-term (> 10 days) 1H3 treatment of infected cultures. After 2 days of infection, macrophages were treated with DMSO, Lamivudine/Lopinavir + Ritonavir (combined antiretroviral therapy), or 1H3 for 10 days, followed by flow cytometry-based analysis of culture viability (LIVE/DEAD staining) and HIV-1 intracellular Gag p24 expression. As shown in Fig. 6e, no significant difference in cell viability between Lamivudine/Lopinavir + Ritonavir, 1H3, or DMSO was observed. Treatment with either Lamivudine/Lopinavir + Ritonavir or 1H3 decreased the frequency of Gag p24^+^ cells reflecting an inhibition of the expanding infection within the culture. However, the Gag p24 mean fluorescent intensity (MFI) of Lamivudine/Lopinavir + Ritonavir-treated HIV-infected cells remained unchanged from the DMSO control, suggesting that the spread of infection was inhibited, but the amount of viral protein expression in cells already infected was unchanged. In contrast, 1H3-treatment not only reduced the frequency of infected cells but also decreased the Gag p24 MFI, suggesting that HIV Gag expression was inhibited in the infected cells. Together, the results from these primary cell systems (human PBMCs, CD4^+^ T cells, and macrophages) using replication competent strains of HIV-1 (BaL and 89.6) indicate that 1H3 inhibits virus replication by altering HIV-1 RNA accumulation to block viral protein expression.

### 1H3 modulates CLK1 and 2 but not CLK3 function

Examination of the kinome profile of 1H3, 2E3, and other pyrazolo[1,5-b] pyridazine derivatives within the PKIS library (using Nanosyn in vitro assays with purified kinases) indicated that compounds within this group which reduced HIV-1 gene expression were also selective inhibitors of the CMGC class of kinases, including the CLKs, in the low nM range [53] (Additional file 1: Fig. S6a). As an initial test of the hypothesis that 1H3 affects SR kinase function, we probed for changes in SR protein abundance upon compound treatment. Analysis of DMSO or 1H3-treated primary HIV_89.6_-infected CD4+ T cell lysates determined that 1H3 induced selective alterations of several SR proteins, reducing SRSF3, SRSF6, and Tra2ß levels by 40 to 50% while increasing SRSF9 expression by almost twofold (Additional file 1: Fig. S4b).

**Fig. 7.**
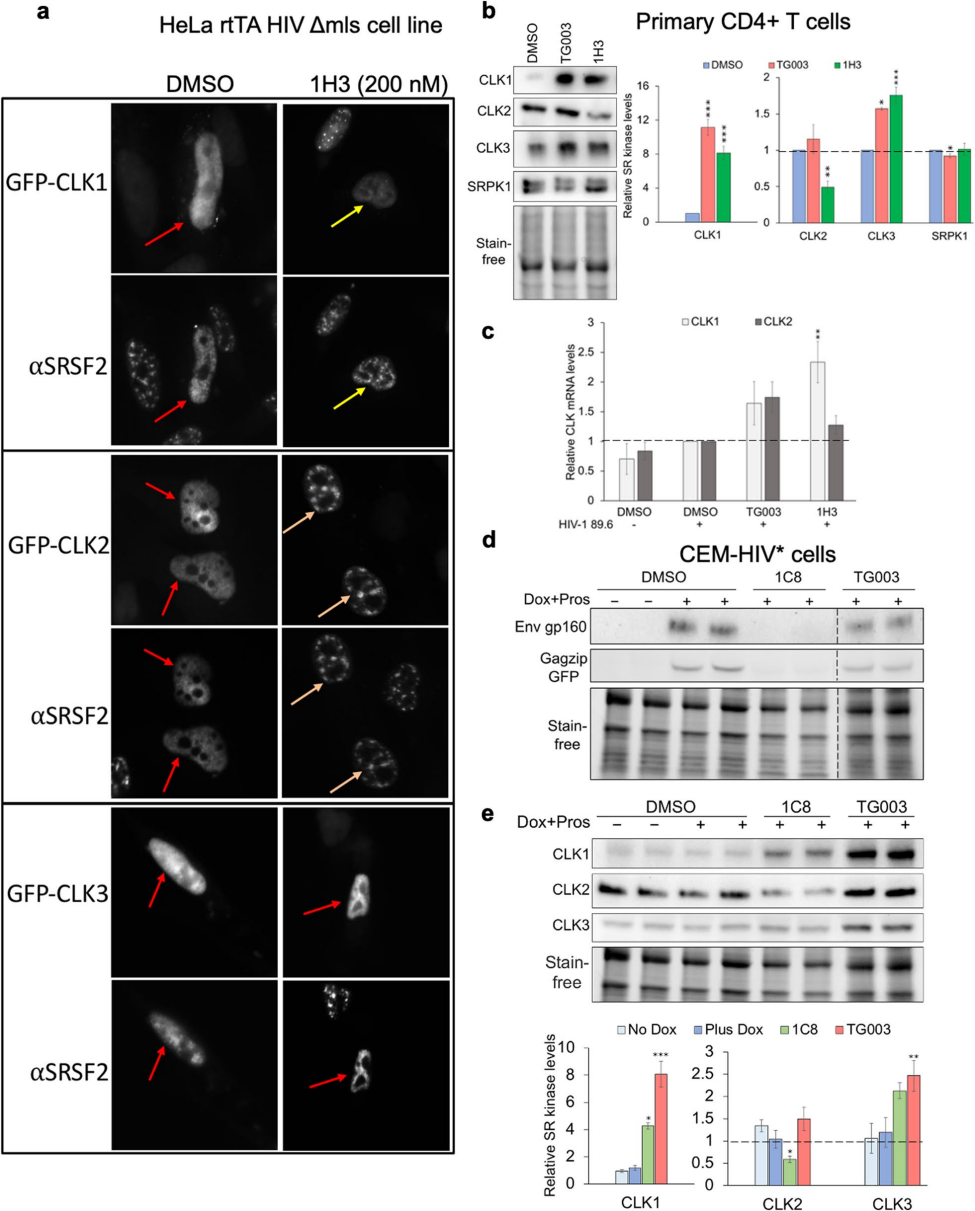
1H3 affects CLK1 and CLK2 function and expression. **a** Effect of compound 1H3 on SRSF2 subcellular distribution upon overexpression of CLKs in HeLa B2 cell line. Cells were transfected with indicated GFP-CLK expression vectors and 48 h post transfection treated with DMSO or 200 nM 1H3 for 24 h, fixed and processed for immunofluorescence. Cells were stained with anti-SRSF2 antibody, a marker for nuclear speckles, and nuclei stained with DAPI. Shown are the representative images of the localization patterns of SRSF2 upon overexpression of CLK1, CLK2, or CLK3. Red arrows indicate loss of nuclear speckles due to CLK overexpression in DMSO or 1H3-treated cells upon CLK3 overexpression. Yellow arrows indicate restoration of nuclear speckles in 1H3-treated cells upon CLK1 or CLK2 overexpression. Images are representative of n = 3 independent experiments. **b**, **c** Healthy donor CD4+ T cells were infected with HIV-1 89.6 then treated with DMSO, 10 µM TG003, or 300 nM 1H3 for 3 d. Cells were harvested and the effect of individual treatments on CLK1, CLK2, CLK3, or SRPK1 **b** protein or **c** mRNA expression determined. Shown on the left in **b** is a representative western blot and, on the right, a summary of assays. Data shown corresponds to results from n > 6 individual patient samples from 2 independent experiments. **d**, **e** 1C8 inhibits HIV-1 gene expression and alters CLK expression. CEM-HIV* cells were treated with 10 µM 1C8 or equivalent volume of DMSO. HIV-1 expression was induced by addition of DOX + prostratin for 24 h. Cells were subsequently harvested, and the lysates analyzed by western blot for **d** HIV-1 Gag and Env expression or **e** expression of CLK1, CLK2, or CLK3. Shown are the representative western blots and below is a graphical summary of the blots across n = 3 independent assays. Band intensity was quantified relative to Dox induced DMSO control and normalized to total protein load using Bio-Rad ImageLab software. *p ≤ 0.05, **p ≤ 0.01, ***p ≤ 0.001

As further evidence that 1H3 acts by affecting SR kinase function, we tested whether the concentration of 1H3 used to suppress HIV-1 expression also inhibits CLK function in cells. In particular, we examined whether 1H3 could reverse CLK-induced disruption of nuclear speckles [36, 62, 63]. HeLa cells expressing individual GFP-tagged CLKs were treated with DMSO or 1H3 and effects on CLK1-3 and SRSF2 (a marker of nuclear speckles) localization examined by immunofluorescence. As shown in Fig. 7a, addition of 1H3 reversed CLK1 and CLK2-induced effects on SRSF2 subnuclear distribution (restoring SRSF2 accumulation to intranuclear speckles), while having no effect on CLK3. 1H3 also altered CLK2 but not CLK1 localization, changing the CLK2 staining pattern from pan-nuclear to colocalization with SRSF2 in nuclear speckles.

The inhibition of CLK1 and CLK2 but not CLK3 activity by 1H3 is similar to the known activity of TG003 [36, 38], raising the question as to why the two compounds differ so dramatically in their ability to suppress HIV-1 replication. To address this question, we examined the effect of these compounds on expression of endogenous CLK1-3 and SRPK1 in primary CD4+ T-cells. Inhibition of CLK1 activity increases its expression due to the enzyme’s role in negatively regulating production of its mRNA [45, 46]. Consistent with being inhibitors of CLK1 activity, addition of either TG003 or 1H3 to primary CD4^+^ T-cells increased CLK1 expression by eight- to tenfold relative to the DMSO control (Fig. 7b) accompanied by a slight (~ 1.5- to twofold) increase in abundance of its mRNA (Fig. 7c). While both TG003 and 1H3 had similar effects on CLK3 (1.5-fold increase) and SRPK1 (no effect) expression, they differed dramatically in their modulation of CLK2 levels. Whereas TG003 addition had limited effect, treatment with 1H3 decreased CLK2 protein levels by 50% (comparable to the level observed with CLK2 shRNA depletion (Fig. 1). 1H3 treatment did not alter CLK2 mRNA levels, suggesting an effect at the post-transcriptional level (Fig. 7c).

As further evidence that compound-induced reduction of CLK2 expression is associated with inhibition of HIV-1, we investigated another compound with potent anti-HIV-1 activity, 1C8. Previously, 1C8 was shown to suppress HIV-1 replication and gene expression (Fig. 7d), an activity linked with its alteration of SRSF10 phosphorylation [35]. More recent in vitro studies (Additional file 1: Fig. S6b) determined that 1C8 inhibits CLK1 function. Consistent with this finding, we observed that the concentration of 1C8 (10 µM) required to fully suppress HIV-1 gene expression in CEM-HIV* cells (Fig. 7d) also increased expression of CLK1 by fourfold and CLK3 by twofold (Fig. 7e). Of note, similar to 1H3, 1C8 treatment reduced CLK2 levels by ~ 50% suggesting that reduced CLK2 kinase expression is associated with inhibition of HIV-1 expression.

## Discussion

Although the current combined antiretroviral therapy (cART) can reduce HIV-1-associated morbidity and prolong survival [64, 65], it is not curative, and is limited by issues of toxicity, treatment adherence, high cost, and emergence of drug-resistant strains [64, 66, 67]. Most importantly, current treatments fail to eliminate latently infected cells established early during acute HIV-1 infection [65]. Furthermore, while the existing drugs target viral entry, reverse transcription, integration, and virion maturation [66], they fail to affect HIV-1 RNA and protein expression in the existing infected cell population. Residual HIV antigens are observed in tissues during cART treatment [68–71], possibly contributing to an increased inflammatory response and the development of co-morbidities [72–79]. Thus, novel approaches to enhance existing therapeutics require identification of host factors or processes critical to the regulation of HIV-1 expression and replication [80]. In this light, the dependency of HIV-1 on host RNA processing machinery offers possibilities of targeting host factors essential for viral gene expression as an alternative strategy to control this infection. Several studies have focused on modulating cellular splicing factors such as SR and hnRNP proteins to alter HIV-1 gene expression [23, 81, 82]. The identification of multiple small molecules (digoxin, ABX464, didehydro-cortistatin A, 8-azaguanine, 5310150, 1C8, 791, 191, filgotinib, GPS491) that inhibit HIV-1 gene expression post-integration by modulating viral RNA processing with limited impact on host cell viability demonstrates the feasibility of this approach [35, 37, 40–43, 83–85].

In this study, we demonstrated that depletion of individual SR kinases has differential effects on HIV-1 gene expression. CLK1 depletion increased HIV-1 gene expression in two T-lymphocyte based cell lines (CEM-HIV*, J-Lat 10.6). In contrast, depletion of CLK2 or SRPK1 dramatically reduced production of HIV-1 structural proteins, Gag and Env. Depletion of CLK3 had little to no effect on viral gene expression. The ability to significantly enhance or reduce HIV-1 gene expression with only a twofold reduction in levels of CLK1 or CLK2, respectively, emphasizes the sensitivity of viral gene expression to the processes regulated by these enzymes. The disparate effects of depleting individual CLKs despite the high degree of sequence similarity (~ 50% overall sequence identity, > 89% sequence similarity in the C-terminal kinase domains) suggests that the functional differences may be due to the divergence present in the N-terminal domain which mediates the interaction with other proteins (Additional file 1: Fig. S7) [62]. Of note, combined depletion of both CLK1 and CLK2 in the CEM-HIV* cell line yielded limited alterations in Gag production (Fig. 3d), indicating that these two kinases act in an antagonistic fashion and that it is the relative abundance of these two kinases (not the absolute levels) which regulate HIV-1 expression. Consequently, changes in their relative expression, as occurs upon CD4^+^ T cell activation, may contribute to the altered capacity of the cell to support HIV-1 expression. Previous work examining the effect of CLK overexpression on HIV-1 gene expression had shown a similar differential effect of individual CLKs; high levels of CLK1 increasing HIV-1 protein production, CLK2 overexpression reducing HIV-1 gene expression, whereas overexpression of CLK3/4 had limited or no effect [36]. The finding that overexpression or depletion of a specific kinase yields a similar effect on HIV-1 gene expression was unexpected yet indicates that the virus is highly sensitive to changes in abundance and activity of these host factors as well as the hyper- or hypophosphorylation of their substrates. The CLK kinases likely function as part of a protein complex. In this instance, depleting the kinase could impair complex assembly whereas excess amounts of the kinase could bind to individual components of the complex and inhibit complex formation to generate the same phenotype. One example of this phenomenon is SRPK1 whose overexpression or depletion promotes cancer by inducing Akt activation [86].

**Fig. 8:**
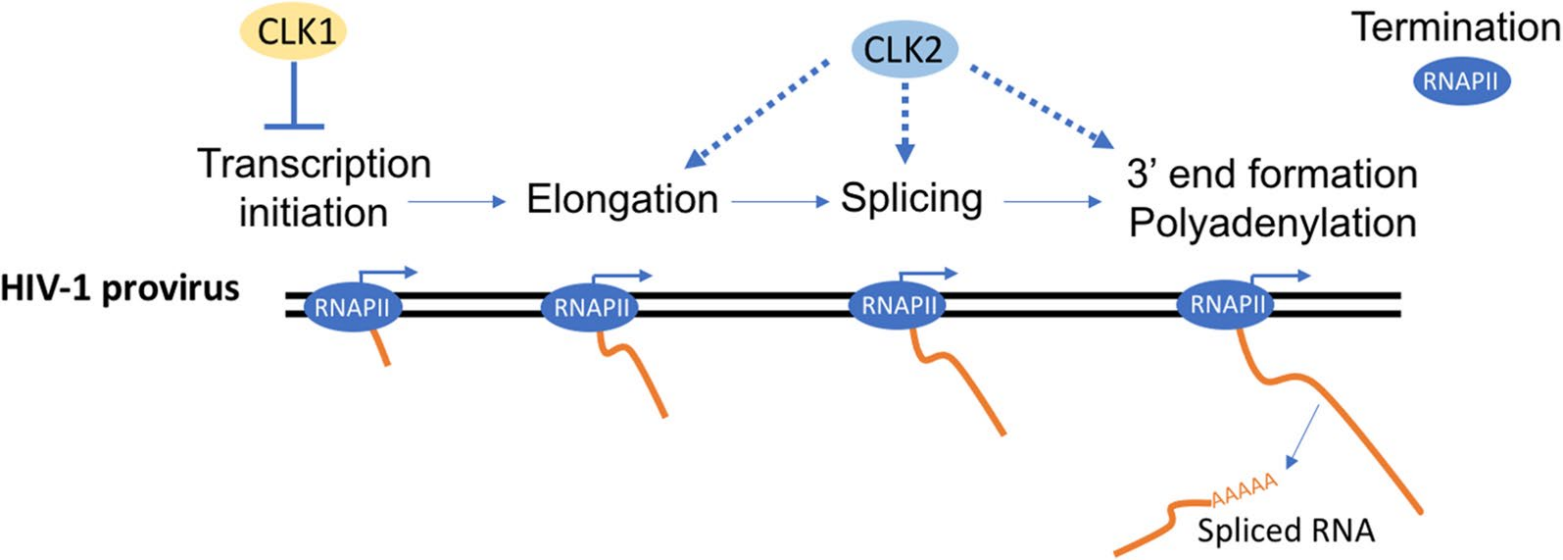
CLK1 and CLK2 act at distinct steps to regulate HIV-1 gene expression. HIV-1 provirus integrated into the host genome is transcribed by the cellular RNA polymerase II (RNAP II). CLK1 acts to suppress the use of the HIV-1 promoter while CLK2 promotes steps in viral RNA synthesis/processing post initiation (elongation, splicing, or 3′ end formation)

Unexpectedly, enhancement or inhibition of HIV-1 gene expression upon depletion of CLK1 or CLK2, respectively, was not correlated with significant alterations in HIV-1 splice site usage or the SR proteins known to regulate their use [23, 87–91] (Additional file 1: Figs. S2a, S4a) but rather with changes in accumulation of viral RNA in general (Fig. 2). Recent studies in primary HIV-1 infected cells identified multiple steps post-transcription initiation that contribute to proviral latency, including blocks to elongation, viral RNA splicing and polyadenylation [48]. Given that HIV-1 TAR RNA levels greatly exceed the level of other viral RNAs (i.e., R-U5-gag) in all the cell systems examined (CEM-HIV* (Additional file 1: Fig. S3), J-Lat 10.6, primary CD4+ T cells) [48, 49], TAR RNA abundance reflects the accumulation of aborted transcripts due to failure of RNA polymerase II to elongate. Consequently, changes in TAR RNA abundance reflect altered transcription initiation [48, 50]. Depletion of CLK2 or CLK3 did not affect TAR RNA abundance despite having different effects on viral US, SS, and MS RNA accumulation (Fig. 3a). Therefore, the reduction in accumulation of HIV-1 mRNAs upon CLK2 depletion suggests effects at steps post-initiation (i.e., polymerase elongation, polyadenylation, RNA stability). Consistent with this interpretation, depletion of CLK2 but not CLK3 reduced abundance of R-U5-gag RNA. In contrast, the increased accumulation of all three classes of viral RNAs as well as TAR and R-U5-gag RNA levels upon CLK1 depletion (Fig. 3a) suggests that CLK1 negatively regulates, directly or indirectly, transcription initiation from the viral promoter. Supporting the hypothesis that CLK1 has a role in regulating HIV-1 transcription initiation, its depletion increased the proportion of cells expressing the HIV-1 provirus, a property consistent with latency reversal (Fig. 3b). Furthermore, CLK1 depletion enhances the activity of several LRAs (prostratin, bryostatin, panobinostat, JQ1, Fig. 3c). Consistent with CLK1 playing an inhibitory role, activation of primary CD4+ T-cells with anti-CD3/anti-CD28, a treatment known to strongly reverse HIV-1 latency by affecting HIV-1 transcript elongation/processing [48], also reduces CLK1 expression. These findings suggest that selective modulation of CLK1 function might be used to augment HIV-1 latency reversal and support a model in which CLK1 acts to suppress use of the HIV-1 promoter while CLK2 promotes steps in viral RNA synthesis/processing post-initiation (working model summarized in Fig. 8). Together, this data indicates very distinct roles for CLK1 and CLK2 in the control of HIV-1 expression, likely the result of the unique set of substrates targeted by each of these kinases. Identifying the host factors mediating these responses will provide greater insight into new approaches to control HIV-1 expression/replication as well as latency.

In support of the hypothesis that targeting of CLK function could prove useful in the development of novel anti-HIV agents, we characterized two CMGC kinase inhibitors from the PKIS library [53] (designated as 1H3 and 2E3) as potent inhibitors of HIV-1 gene expression and replication. Various SR kinase inhibitors have been examined for their ability to inhibit HIV-1 expression and replication, including TG003 (CLK1, 2, and 4 inhibitor), chlorhexidine (CLK2, 3, and 4 inhibitor), and SRPIN340 (SRPK1 and 2 inhibitor) [36, 37, 92],but only chlorhexidine effectively suppressed HIV-1 gene expression in cell-based assays [36, 37]. However, the toxicity of chlorhexidine precludes its use as a therapeutic. In contrast, 1H3 potently inhibited (EC_50_ ~ 50 nM) HIV-1 replication and expression in multiple cell-based assays (including primary cells) with limited cell toxicity by reducing viral Gag, Env, Tat, and Nef expression and viral RNA accumulation. The reduction of TAR RNA levels following compound treatment (Fig. 5d) suggests inhibition of initiation from the HIV-1 promoter. Tests in primary cells infected with replication competent HIV-1 strains confirmed 1H3’s ability to strongly inhibit virus replication (comparable to AZT and combined antiretroviral therapy Lamivudine/Lopinavir + Ritonavir) without cytotoxicity at a dose similar to that used to suppress the virus gene expression in transformed cells (Fig. 6). Of note, unlike Lamivudine/Lopinavir + Ritonavir, addition of 1H3 also reduced the intracellular levels of Gag (demonstrated by decreased Gag MFI) in infected macrophages, consistent with reduced viral RNA accumulation. A reduction in viral antigen load in infected cells may be advantageous if the presence of such antigen contributes to the chronic immune activation present during this infection [93]. In vitro evaluation of 1H3’s inhibition of a panel of purified host kinases (as measured by Nanosyn assay [53]; Additional file 1: Fig. S6a) and its ability to reverse CLK-induced disruption of nuclear speckles in cells (Fig. 7a) confirmed the compound’s ability to inhibit CLK1 and CLK2 but not CLK3 function. However, the similarity in the spectrum of CLKs affected by 1H3 and TG003 but marked difference in effect on HIV-1 was initially puzzling. The increased levels of CLK1 upon addition of either TG003 or 1H3 is consistent with the known negative feedback of CLK1 on production of its mRNA (Fig. 7b, c) [94, 95]. But, in contrast to TG003, 1H3 addition decreased CLK2 expression (~ 50% of control, Fig. 7b), comparable to the reduction achieved in shRNA depletion studies (Fig. 1) that also abrogated HIV-1 gene expression. The increase in CLK1 and reduction in CLK2 levels upon addition of 1C8 (Fig. 7e), another inhibitor of HIV-1 replication and modulator of viral RNA processing, suggests a similar underlying pattern in response. Consequently, although both 1H3 and TG003 inhibit CLK2 activity (as measured by their reversal of the effects of CLK overexpression on nuclear speckles (Fig. 7a, [36]), it is the modulation of CLK2 abundance that best correlates with a compound’s ability to block HIV-1 expression. While 1H3/2E3 addition mimics several of the effects of CLK2 depletion in HIV-1 protein expression, the differential effect on TAR RNA abundance suggests distinct mechanisms of action.

In addition to their well-documented role in regulating splice site usage, SR proteins participate in multiple stages of RNA metabolism including transcription initiation/elongation, polyadenylation, transport to the cytoplasm, and translation [5, 14, 18, 96–98]. SR protein localization and activity is sensitive to its state of phosphorylation and individual SR kinases are known to modify them to different extents [12, 34, 52, 63, 96, 98–100]. Despite the ability of all the SR kinases evaluated (CLK1-3, SRPK1) to modify SR proteins in vitro [34], depletion of individual SR kinases, their altered expression upon T-cell activation, or inhibition upon compound treatment (1H3) yielded only limited and selective changes in SR protein abundance or phosphorylation (as indicated by changes in protein mobility). Furthermore, the pattern of changes varied despite similar effects on virus expression. Differences between 1H3 and individual CLK depletion (see Additional file 1: Fig. S4a, b) are not unexpected since shRNA reduced the level of only the targeted enzyme (the remainder being free to modify substrates) while 1H3 impacts the function/expression of several SR kinases and the loss of CLK enzymatic function is likely more extensive. The SR proteins affected by CLK1/CLK2 depletion, T cell activation, or 1H3 treatment are known to impact HIV-1 gene expression [23, 87–91]. Of the changes detected, both CLK2 depletion and 1H3 addition reduced SRSF6 levels, known to regulate both HIV-1 RNA splicing and translation [22, 87, 90, 101]. Modulation of SRSF6 expression affects generation of Tat mRNA [87] and its depletion negatively impacts HIV-1 gene expression (data not published). The loss of Tat upon 1H3 treatment/CLK2 depletion (Figs. 1 and 6) would result in the loss of Tat’s stimulation of transcription elongation from the viral promoter [1], accounting for the reduced HIV-1 RNA accumulation seen. Similarly, CD3/CD28 activation versus 1H3 treatment in the context of primary CD4^+^ T cells had opposing effects on SRSF3 and Tra2ß levels, modulation of either resulting in marked changes in HIV-1 gene expression [37, 89]. Consequently, either HIV-1 expression is highly sensitive to the perturbations of these SR proteins or the modification of host factors other than SR proteins by individual CLKs [102] underly the responses observed.

## Conclusion

Taken together, our observations indicate that SR kinases have distinct roles in the regulation of HIV-1 expression and suggest that efforts to further refine the therapeutic targeting of HIV-1 expression, to either reverse (CLK1 inhibition) or re-enforce (CLK2 inhibition) latency, might focus on enhancing the specificity of CLK1 or CLK2 inhibitors by exploiting the small differences among these kinases. Although the CLK kinase domains are structurally similar to one another, a closer examination of the active sites revealed notable difference in the pocket size and electrostatic surface charge distributions [32]. These differences might explain the selective inhibitory effect of the different compounds on the CLKs. The active site of CLK1 is somewhat narrow with a negatively charged patch. In CLK3, a protruded Lys248 makes entry of substrate unfavorable into the active site [32]. CLK2 has the weakest charge distributions among the three CLKs, and Val326 in the active site favors weak hydrophobic interactions [32] which might favorably assist interaction with the benzyl ring of the compound. These differences in the binding pocket among the CLKs may permit the generation of compounds with a more selective effect on CLK1 or CLK2 function. Consistent with the hypothesis that HIV-1 RNA processing can be a therapeutic target, we show that CMGC kinase inhibitors (1H3/2E3) effectively suppress viral gene expression/replication at nanomolar dose with limited impact on host cell viability. More extensive characterization of the changes in host protein phosphorylation affected by CLK depletion or 1H3/2E3 treatment may provide a framework to investigate novel factors regulating HIV-1 gene expression.

## Materials and methods

### shRNA plasmids, lentivirus production, and HIV-1_89.6_ production

Plasmids expressing shRNAs targeting CLK1, CLK2, CLK3, and SRPK1 were generously provided by Dr. Jason Moffat (University of Toronto). The shRNA sequences were cloned into pLKO.1 expression vector that drives shRNA expression from a human U6 promoter and contains a puromycin selection marker [103]. Sequences of shRNAs used are shown in Additional file 1: Table S2. To generate shRNA lentiviruses, 2.5 million HEK293T17 cells (Cat# ATCC CRL-11268) were transfected in 10 cm dish with three-plasmid lentiviral packaging system comprising of pLKO shRNA vector (6 µg), vesicular stomatitis virus G protein (VSV-G) envelope plasmid (0.6 µg), and packaging plasmid as pAX2 (5.4 µg) [103–105]. Cells were transfected using polyethyleneimine (PEI, Sigma) in Opti-MEM (Gibco) and serum free Iscove’s modified Dulbecco’s medium (IMDM, Wisent Corp.). After 5 h of transfection, the culture medium was replaced with IMDM complete medium [IMDM supplemented with 10% v/v fetal bovine serum (FBS), 1% Penicillin/Streptomycin (P/S), 0.2% Amphotericin B, Wisent Corp.]. Supernatant containing lentivirus particles was harvested 72 h post-transfection and filtered through 0.45 µm filter to remove cell debris. Virus aliquots were stored at − 80 °C until further use.

To generate HIV-1_89.6_ (an X4R5 macrophage-tropic primary isolate of HIV-1) virus stocks, 25 million HEK293T cells were plated in a T225 cm^2^ flask overnight in 50 mL of D10 media: Dulbecco’s Modified Eagle Medium (DMEM) with high glucose and pyruvate (ThermoFisher, Cat#1995065) containing 10% FBS, 1% Penicillin/Streptomycin, and 2 mM l-glutamine. 50 µg of HIV-1_89.6_ proviral plasmid DNA (NIH AIDS Reagent Program, Cat#3552; contributed by Dr. Ron Collman) was prepared in 6.5 mL of serum-free DMEM and then mixed with 200 µg of PEI. After 10 min, the DNA:PEI mixture was added to the cells, for which the original D10 media was exchanged for 50 mL of fresh media. Culture supernatants were collected 72 h post-transfection, centrifuged to pellet debris, filtered through a 0.45 µM filter, concentrated with PEG-*it* Virus Precipitation Solution (System Biosciences, Cat#LV825A-1) as per the manufacturer’s instructions, resuspended in 100-fold less volume of media and stored at − 80 °C until further use.

### Cell lines

CEM-T4 HIV GagzipGFP (CEM-HIV*) cell line was generated through transduction of CEM-CD4+ T (NIH AIDS Reagent Program Cat #117) cells with the proviral construct, HIV rtTA GagzipGFP (Fig. 1a), a derivative of previously described Dox inducible HIV-1 provirus, rtTA HIV∆mls [42, 45]. HIV rtTA GagzipGFP construct was generated by replacing the Spe1/Mls1 fragment of rtTA HIV∆mls with the same fragment from pNL4-3 GagzipGFP (provided by L. Parent, Penn State College of Medicine) in which the nucleocapsid (NC) portion of Gag was replaced with the leucine zipper of the human cAMP responsive element binding (CREB) protein and GFP was inserted in-frame with Gag. This insertion deleted PR and RT sequences up to the second Mls1 site of the HIV-1 LAI rtTA. To generate virus particles, HEK293T cells were transfected with HIV rtTA GagzipGFP, along with pAX2 and VSV-G vectors. Supernatants were filtered and used to infect CEM-T4 cell lines. Cells were then selected by fluorescence-activated cell sorting (FACS) for Dox-dependent Gag-GFP expression and clones established by limiting dilution. Although the cells were FACS sorted several times to select for GagzipGFP expression in the presence of Dox, a significant fraction of the cells did not induce Gag expression after subsequent culture in the absence of Dox followed by reactivation revealing establishment of a state of latency in the cells. A protein kinase C (PKC) activator, prostratin (Sigma-Aldrich), at a concentration of 2.56 µM along with Dox (4.5 µM) was used to induce HIV-1 expression from this latent cell line. HeLa rtTA HIV GagGFP (HeLa C7) cell line used for screening of GlaxoSmithKline (GSK) published kinase inhibitor set (PKIS) library has been previously described [54]. HeLa rtTA HIV∆mls (HeLa B2) cell line used for CLK overexpression assay has been previously described [36, 42]. J-Lat 10.6 clone, a Jurkat-based T cell line [56], was obtained from NIH AIDS Reagent Program (#9849). CEM-HIV* and J-Lat 10.6 cells were cultured in Roswell Park Memorial Institute (RPMI) medium (Wisent Corp., RPMI 1640) supplemented with 10% v/v FBS, 1% P/S, and 0.2% Amphotericin B. HeLa B2 and HeLa C7 cells were maintained in IMDM complete medium. All the cell lines were maintained at 37 °C in a humidified incubator with 5% CO_2_.

### Primary cell preparation

#### CD4+ T cells preparation for activation assay

Human peripheral blood mononuclear cells (PBMCs) were isolated from buffy coats of healthy (HIV-uninfected) human donors using Ficoll-Paque™ Plus (Cytiva SE-75184 Uppsala, Sweden) following manufacturer’s protocol. Donor blood samples for this assay were obtained from Canadian Blood Services and St. Michael’s Hospital located in Toronto, Ontario, Canada. Informed consent was obtained from participants in accordance with the guidelines for conduct of clinical research at the University of Toronto, Toronto, Ontario, Canada. CD4+ T cells were isolated on the same day from freshly prepared PBMCs using an immunomagnetic negative selection isolation kit, EasySep Human CD4+ T Cell Isolation Kit (STEMCELL Technologies, Cat#17952) as per manufacturer’s instructions. Following isolation of CD4+ T cells, 5 million cells were plated onto each well of a 12-well non-treated TC dish that was coated overnight with 2 μg/mL of anti-CD3 antibody (Ultra-Leaf™ Purified anti-human CD3 Clone: OKT3, BioLegend, Cat#317326) and washed with 1XPBS, in the presence of RPMI-1640 complete medium containing 2 μg/mL of anti-CD28 antibody (Fitzgerald, Batch#0979) and 10 ng/mL IL-2 (Sigma-Aldrich, Cat#I7908-10KU). Control CD4+ T cells (untreated) were plated on the same 12-well dish coated with only PBS, washed, and plated in the presence of complete RPMI 1640 medium (without anti-CD28 or IL-2). Cells were then harvested at 24 h, 48 h, 4 d, or 6 d post-treatment for protein analysis by western blots and RNA analysis by RT-qPCR assay.

#### Cell preparation for infection assay

PBMCs were isolated by leukapheresis (Spectra apheresis system; Gambro BCT) from healthy (HIV-uninfected) donors. Buffy coats were collected using Ficoll-Paque Plus (Amersham Biosciences) following manufacturer’s protocol. Informed consent was obtained from participants in accordance with the guidelines for conduct of clinical research at the University of Toronto and St. Michael’s Hospital, Toronto, Ontario, Canada. Prepared PBMCs were frozen at − 80 °C in 90% v/v FBS and 10% DMSO for subsequent experimentation.

Macrophages and CD4^+^ T cells were prepared as previously described [106]. Buffy coats from anonymous HIV-uninfected healthy donors were acquired from the Massachusetts General Hospital Blood Bank. All human subjects gave written, informed consent for use of their blood products for research purposes. Monocytes were isolated from frozen PBMCs using the EasySep Human CD4 Positive Selection Kit II (STEMCELL Technologies, Cat#17858) as per manufacturer’s instructions. CD4^+^ T cells were enriched from the remaining CD14-depleted PBMCs using the EasySep Human CD4^+^ T cell Isolation Kit (STEMCELL Technologies, Cat#17952) as per the manufacturer’s instructions. Monocytes were matured into macrophages over 7 days in 6-well low attachment plates (2 million/well—Sigma, Cat#CLS3473) in the presence of 50 ng/mL recombinant GM-CSF (BioLegend, Cat#572904) and 50 ng/mL recombinant M-CSF (BioLegend, Cat#574806) in R10 Media: RPMI-1640 (Sigma-Aldrich, St. Louis, MO) containing 10% Certified FBS (ThermoFisher, Cat#16000044), 1% P/S, 2 mM l-glutamine, and 10 mM HEPES (Corning Inc). Certified FBS lots were chosen based on low endotoxin and their ability to mature macrophages that yielded efficient levels of HIV infection. Half of the R10 media containing fresh GM-CSF/M-CSF was exchanged on the 4th day of maturation. Successful 7-day maturation was assessed via spreading of the cells onto the surface of the plate. In parallel, CD4^+^ T cell targets were plated onto 24-well non-treated TC plates (0.8 million/well, Sigma, Cat#CLS3738), which were coated overnight with 2 μg/mL of anti-CD3 antibody (BioLegend, Cat#317326) and then washed, in R10 media containing 2 μg/mL of anti-CD28 antibody (BioLegend, Cat#302934) and 10 ng/mL IL-2 (R&D Systems, Minneapolis, MN, Cat#202-IL-500). After 4 days of activation, the cells were removed from the plate, washed in R10 media, and rested for an additional 3 days at an initial concentration of approximately 0.5 million cells per mL before infection.

### shRNA depletion in cell lines

Depletion assays were done in 6-well plates. Briefly, 4.0 * 10^5^ cells/well were seeded for each cell line tested. Prior to infection, shRNA viral titers were determined with alamarBlue™ Cell Viability Reagent (ThermoFisher Scientific) following puromycin selection. Cells were infected with shRNA lentivirus in complete medium containing 21.4 µM polybrene for 24 h. Following infection, cells were replenished with fresh medium containing the selection agent, puromycin (4.24 µM) for 72 h. After selection, HIV-1 gene expression was induced by addition of Dox (4.5 µM) in the context of HeLa B2 cell line, Dox (4.5 µM) and prostratin (2.56 µM) in CEM-HIV*, or prostratin (2.56 µM) only in the J-Lat 10.6 cell line. Cells were harvested 24 h following induction and processed for western blotting and RNA analyses.

### Screening of GSK published kinase inhibitor set (PKIS) I and II for anti-HIV-1 activity

PKIS I and II libraries were kindly provided by GSK LCC (Research Triangle Park, NC USA) [55]. From the library, 670 compounds were screened for their effect on HIV-1 Gag expression in HeLa C7 cell line [54]. For initial screening, the compounds were assayed at a final concentration of 10 µM. Briefly, 2 × 10^4^ cells/well were seeded on 96 well plate in IMDM complete medium. Cells were incubated with inhibitor compound and HIV-1 gene expression induced by addition of 4.5 µM Dox. 1% DMSO-treated cells incubated with or without Dox served as positive and negative controls, respectively. After 24 h, media was removed, and cells fixed in 3.7% formaldehyde in PBS for 30 min at room temperature. Following fixation, formaldehyde was removed, replaced with 1× PBS, and plates stored at 4 °C prior to scanning. Plates were imaged for GagGFP expression using Typhoon 9400 (Amersham Biosciences) at 488 nm excitation and 526 nm emission filters. Only those compounds that exhibited no visual changes in cell morphology (as observed by phase contrast microscopy), no reduction in cell viability, and showed a significant reduction of Gag-GFP signal were analyzed further.

### Compound treatment assays

#### Cell viability assay with 1H3 (GW801372X) and 2E3 (W806290X)

Relative cell viability upon treatment with 1H3 **(**GW801372X) or 2E3 **(**W806290X) was assessed by using alamarBlue™ Cell Viability Reagent (ThermoFisher Scientific). Briefly, HeLa C7 cells were seeded at a density of 20,000 cells per well of a 96 well plate and treated with a range of compound concentrations (up to 10 µM). After 24 h, culture medium containing alamarBlue was added and incubated at 37 °C and 5% CO_2_ for 1 to 3 h. Fluorescence was monitored at an excitation wavelength of 530 nm and 590 nm emission wavelength using BioTek Cytation5 plate reader.

#### Effect of compounds 1H3 and 2E3 on HIV-1 gene expression and host SR protein abundance in cell lines

Compound treatment assays were performed as previously described [40, 42]. In the context of adherent cells, cells were seeded at 70% confluence in IMDM complete medium on 6 well plate. The following day, culture medium was removed and replaced with fresh medium and compounds at desired concentration (final concentration of 200 nM and 100 nM of 1H3 and 2E3, respectively). Control wells contained 1% DMSO with or without Dox. For suspension cells, one million cells/well were pelleted and resuspended in RPMI complete medium and treated with compound or DMSO and induced on the same day. Cells were harvested 24 h post-induction for western blotting and RNA analyses.

#### Compound treatment assays in primary cells

To examine the effect of compound 1H3 on viral replication, stored PBMCs were thawed, washed with RPMI 1640 complete medium and cultured in RPMI 1640 complete medium containing 2 μg/mL of PHA-L (Sigma-Aldrich) and 20 U/mL of IL-2 (BD Pharmingen) for 72 h, as described previously [42]. Subsequently, cells were counted, and a portion placed in another tube for uninfected control treatments. The remaining PBMCs were resuspended in media containing HIV-1 BaL at a multiplicity of infection (MOI) of 10^–2^ and infected by spinoculation. Following spinoculation, cells were washed twice with RPMI 1640 complete medium and resuspended to a concentration of 5 × 10^5^ cells/mL in complete RPMI 1640 containing 20 U/mL of IL-2. Compounds were added to infected PBMCs or uninfected control PBMCs. Azidothymidine (AZT, Sigma-Aldrich) was used as control treatment at a final concentration of 3.74 μM. On day 4 post infection, culture medium was replenished with the compounds and IL-2 in fresh complete RPMI 1640. Aliquots of culture media were harvested on 2, 4, 6 and 8-day post infection, stored at − 20 °C, and virus replication assessed using HIV-1 p24 antigen ELISA kits purchased from Frederick National Laboratory for Cancer Research (Leidos) according to manufacturer’s protocol. Cells were also harvested to assess percent cell viability by trypan blue exclusion using Glasstic slides (Kova) and relative percent cell viability in compound treated sample versus DMSO-control treated sample calculated as described previously [42].

For the assay that employed primary CD4+ T cells, activated CD4+ T cells were spinoculated with HIV-1_89.6_ for 1 h followed by an additional 3-h incubation at 37 °C. Virus was aliquoted onto 5 million cells per treatment condition, at 1 million cells per well of a 96 well flat bottom plate in 50 μL of R10 media with 10 ng/mL IL-2 (R&D Systems). The volume of virus added to each well of CD4+ T cells was determined based on the viral tittering of each stock that yielded saturated levels of infection, which typically ranged from ~ 25 to 50%, depending on the donor. After infection, the cells were pooled, washed once in R10 media to remove the virus, and then plated out in 6 well plates at 5 million cells/well in 4 mL of R10 + 10 ng/mL IL-2+ inhibitor (1% DMSO, 300 nM 1H3, or 10 μM TG003). After 3 days, the cells were harvested, 10% was used for flow cytometry analysis, 45% was used for western blot analysis (lysed at 5 million cells per 100 µL of RIPA Buffer) and 45% was used for the RNA analysis (lysed in 300 µL of Aurum Total RNA Lysis kit).

For the assays using infected macrophages, media was removed from 7-day differentiated macrophages, and fresh macrophage media (R10 containing GM-CSF/M-CSF) was added back to each well. The volume of virus added to each well of macrophages was determined based on the viral titering of each stock that yielded saturated levels of infection, typically ranging from ~ 10 to 50%, depending on the donor. The cells were incubated for 6 h at 37 °C, followed by removal of the virus and addition of the original conditioned media diluted 1-in-2 with fresh macrophage media. After 2 days of infection, the cells were pooled and set up for long-term cultures. Briefly, the macrophages were washed in PBS and lifted from the low attachment plates using Cell Dissociation Buffer (ThermoFisher, Cat#13151014) for 10 min at 37 °C, pooled and plated at 250,000 cells per well of a 24-well low attachment plate (Sigma-Aldrich, Cat#CLS3471) in 500 μL of macrophage media with either DMSO, Lopinavir/Ritonavir- 53 nM and Lamivudine—1.5 μM), or 1H3 (300 nM). At day 4 post-treatment, half of the media was exchanged for new media containing fresh preparations of the inhibitors. Day 10 post-treatment, the macrophages were lifted from the plate and transferred to a 96-well V-bottom plate.

For assays that examined relative SR protein expression in uninfected primary CD4^+^ T cells, after 7 days of activation, the cells were treated with a DMSO control or 300 nM of 1H3 for 72 h in R10 media, and then harvested for western blots.

#### Effect of 1H3 on CLK activity/expression

To determine if compound 1H3 modulates CLK function, its ability to prevent disruption of SRSF2 nuclear speckling pattern upon CLK overexpression was examined as previously described [36]. HeLa B2 cells were seeded on coverslips at ~ 60–70% confluence in 6 well plate in IMDM complete medium. Cells expressing GFP-tagged CLK1, CLK2, or CLK3 (kindly provided by J. Bell, University of Ottawa) were induced with Dox for 24 h in the presence of 1H3 (200 nM) or DMSO control and fixed with 4% paraformaldehyde in PBS. Cells were permeabilized with 1% Triton X-100 in PBS followed by blocking in 3% BSA in PBS. Coverslips were then stained with mouse anti-SC35 (SRSF2) antibody (Sigma-Aldrich) followed by incubation with Texas Red-labeled donkey anti-mouse antibody (Jackson ImmunoResearch). Cells were DAPI stained to detect nuclei prior to mounting. Images were captured on Leica DMR microscope at 630× magnification. To measure effects of compounds on SR kinase expression, DMSO or compound-treated lysates were run on 10% TGX stain-free gels (Bio-Rad) and, following transfer to PVDF, blots were probed with antibodies to CLK1, CLK2, CLK3, or SRPK1. TG003 and 1C8 were each used at a concentration of 10 μM.

### Western blot analyses

Protein extracts were prepared from cells using RIPA buffer (50 mM Tris–HCl pH 7.5, 150 mM NaCl, 1% NP-40, 0.5% sodium deoxycholate, 0.1% SDS) supplemented with Halt Phosphatase Inhibitor (ThermoFisher). The extracts were fractionated on 10% TGX acrylamide stain-free gels (Bio-Rad) or 14% SDS-PAGE. Stain free gels were directly imaged on ChemiDoc MP Imager (Bio-Rad) prior to transfer to PVDF to measure total protein levels which served as loading control [107]. Gels were directly imaged to measure GagGFP and GFP expression in CEM-HIV* and J-Lat 10.6 cells, respectively. Following imaging, proteins were transferred to PVDF (0.45 µM, Bio-Rad) using Trans-blot Turbo Transfer System (Bio-Rad). Immunoblots were blocked in 5% milk-TBS-T (5% milk, 0.05% Tween-20, 1× TBS) or 5% BSA-TBS-T (5% BSA, 0.05% Tween-20, 1× TBS) for 1 h at room temperature prior to incubating in primary antibodies. After primary antibody incubations, blots were washed in 1× TBS-T and incubated in appropriate secondary antibody for an hour at room temperature. Following subsequent washes, blots were developed using Clarity Western ECL substrate (Bio-Rad) and imaged on ChemiDoc MP Imager (Bio-Rad). Band intensity was quantified relative to control in the experiment (shControl or DMSO) and normalized to the corresponding bands of the loading control (total protein for 10% gels and GAPDH for 14% gels) using ImageLab software (Bio-Rad). Details of all the primary antibodies used in this study are shown in Additional file 1: Table S3.

### RNA analyses

#### Quantitation of HIV-1 mRNA levels

Samples for quantitation of HIV-1 mRNA levels were processed using Aurum Total RNA Lysis kit (Bio-Rad) as per manufacturer’s instructions with the addition of Turbo DNase (Ambion). Purified RNA (0.5–1 µg) was reverse transcribed using M-MLV reverse transcriptase (Invitrogen) as previously detailed [42]. cDNA reactions (20 µL) were diluted to 100 µL and quantified for HIV-1 mRNA levels by quantitative PCR (qPCR) using CFX384 Touch Real-Time PCR Detection System (Bio-Rad). Standard curve method was used for the quantitation of viral mRNA levels, normalized to the housekeeping gene, ß-actin, and expressed relative to control treatment (sh control for the knockdown or DMSO for compound). Each reaction was set up as follows in 384 qPCR well plate: 1 µL of Taq DNA polymerase (5 U/µL, abm), 1 µL of 10× PCR buffer with Mg^2+^ (abm), 0.2 µL of 10 mM dNTP mix (Bio Basic Canada Inc. #D0046C), 1 µL of 10× SYBR Green I (Sigma-Aldrich), and 0.2 µL of each 5′ and 3′ primers (10 µM) in a total reaction volume of 10 µL. The sequences of forward and reverse primers used for quantitation of HIV-1 unspliced (US), singly spliced (SS), multiply spliced (MS), and TAR RNA levels are: US-5′ GACGCTCTCGCACCCATCTC 3′ and 5′ CTGAAGCGCGCACGGCAA 3′; SS-5′ GGCGGCGACTGGAAGAAGC 3′ and 5′ CTATGATTACTATGGACCACAC 3′; MS-5′ GACTCATCAAGTTTCTCTATCAAA 3′ and 5′ AGTCTCTCAAGCGGTGGT 3′; TAR-5′ GTCTCTCTGGTTAGACCAG 3′ and 5′ TGGGTTCCCTAGYTAGCC 3′. The sequences of the forward and reverse primer set for the reference genes are: ß-actin 5′ TGACGTGGACATCCGCAAAG 3′ and 5′ CTGGAAGGTGGACAGCGAGG 3′; PUM1 5′ TGAGGTGTGCACCATGAAC 3′ and 5′ CAGAATGTGCTTGCCATAGG 3′. The qPCR cycling conditions were as follows: activation at 95 °C for 3 min followed by 40 cycles of denaturing at 95 °C for 15 s, annealing at 55 °C for 25 s, and extension at 68 °C for 30 s. The melting curve protocol followed with 15 s at 95 °C and then 15 s each at 0.2 °C increments between 55 and 95 °C. Melting and standard curves were generated by the CFX Maestro Software (version 1.1, Bio-Rad). Analysis of TAR RNA levels was performed essentially as previously described [48], with the exception that classical qPCR was used for quantification.

#### Analysis of splice site selection within HIV-1 MS RNA

The effect of individual kinase depletion or compound treatment on splice site selection within HIV-1 MS RNA was performed by RT-PCR [108] followed by running the amplified PCR products on 7% polyacrylamide gel (PAGE). cDNA samples were prepared as described in “Quantitation of HIV-1 mRNA levels” section. The PCR reactions were set up as follows: 5 µL of diluted cDNA, 2 µL of 10× Thermopol buffer (NEB #B9004S), 0.5 µL of 10,000 units AdvanTech Taq DNA Polymerase (#AD100-11103), 2 µL of 2.5 mM dNTP, 1 µL of each 5′ and 3′ primers (10 µM) in a total reaction volume of 20 µL. The forward and reverse primer pairs used to amplify HIV-1 MS RNAs are as follows: 5′ CTGAGCCTGGGAGCTCTCTGGC 3′ and 5′ CCGCAGATCGTCCCAGATAAG 3′. The thermocycler cycling conditions were as follows: initial activation at 94 °C for 2 min followed by 27 cycles of denaturing at 94 °C for 60 s, annealing at 57 °C for 60 s, and extension at 68 °C for 60 s and final extension at 68 °C for 5 min followed by hold at 4 °C. To the amplified products, 6 µL of 6× Purple Gel Loading Dye (NEB #B7024S) was added and 10 µL of reaction run onto 7% PAGE gel (10× TBE (Tris–borate EDTA), 30% acrylamide, 400 µL 10% ammonium persulfate, and 30 µL TEMED) at 250 V for 3 h. The gel was then soaked onto Ethidium bromide solution prepared in 10× TBE for 5 min and visualized in Chemidoc MP Imager (Bio-Rad).

#### Analysis of CLK1 and SRPK1 mRNA levels in untreated versus activated primary CD4+ T cells

Samples for quantification of CLK1 and SRPK1 mRNA levels were processed as discussed in “Quantitation of HIV-1 mRNA levels” section. Purified RNA (0.2 µg) was reverse transcribed using M-MLV reverse transcriptase (Invitrogen) as previously detailed [42]. cDNA reactions (20 µL) were diluted to 100 µL and quantified for CLK1 or SRPK1 mRNA levels by quantitative PCR (qPCR) using CFX384 Touch Real-Time PCR Detection System (Bio-Rad). Standard curve method was used for the quantitation of viral mRNA levels, normalized to the housekeeping gene, ß2-microglobulin (ß2M), and expressed relative to untreated sample. Each reaction was set up as follows in 384 qPCR well plate: 5 µL of SsoAdvanced™ Universal SYBR Green Supermix (Bio-Rad, Cat#1725271), 0.1 µL of each 5′ and 3′ primers (10 µM), and 2 µL of cDNA template in a total reaction volume of 10 µL. The sequences of forward and reverse primer pairs used for quantitation of CLK1 and SRPK1 mRNA levels are: CLK1-5′ AGAGACCATGAAAGCCGGTAT 3′ and 5′ CATGTGAACGACGATGTGAAGT 3′; and SRPK1-5′ ATGGAGCGGAAAGTGCTTG 3′ and 5′ GAGCCTCGGTGCTGAGTTT 3′. The sequences of the forward and reverse primer set for the reference gene ß2M are: 5′ CTATCCAGCGTACTCCAAAG 3′ and 5′ GAAAGACCAGTCCTTGCTGA 3′. The qPCR cycling conditions were as follows: activation at 95 °C for 3 min followed by 40 cycles of denaturing at 95 °C for 30 s, annealing at 55 °C for 30 s, and extension at 72 °C for 30 s. The melting curve protocol followed with 15 s at 95 °C and then 15 s each at 0.2 °C increments between 55 and 95 °C. Melting and standard curves were generated by the CFX Maestro Software (version 1.1, Bio-Rad). CLK1 or SRPK1 mRNA levels were normalized to ß2M and mean mRNA levels were expressed relative to untreated control.

#### Quantification of CLK1 and CLK2 mRNA levels in HIV-1_89.6_ infected primary CD4+ T cells

Samples for quantification of CLK1 and CLK2 mRNA levels were processed as discussed in “Analysis of CLK1 and SRPK1 mRNA levels in untreated versus activated primary CD4+ T cells” section. CLK1 primer pairs used for quantitation of CLK1 mRNA are shown in “Analysis of CLK1 and SRPK1 mRNA levels in untreated versus activated primary CD4+ T cells” section. The CLK2 primer pairs used for the quantification of CLK2 mRNA levels are: 5′ GGGGAGTTACCGTGAACACTA 3′ and 5′ CGTGTCCGGTCACTACTACTTG 3′. Mean mRNA levels were normalized to ß-actin and expressed relative to HIV-1_89.6_-infected DMSO control.

#### In vitro CLK1 inhibition assays

Recombinant human CLK1 (50 ng, C57-11G, SignalChem Biotech Inc. Canada) was incubated with DMSO or a range of serial dilutions of 1C8 in kinase buffer (40 mM Tris–HCl pH 7.5, 25 mM MgCl_2_, 0.1 mg/mL BSA and 0.25 mM DTT) containing 50 µM of ATP (Promega, Madison, WI, USA). The 5 µL kinase reaction performed in 384-wells Bio-Rad white plate was incubated for 30 min at 30 °C. The reactions were stopped by adding 5 μL of ADP-Glo reagent (Promega, V6930) and incubated at room temperature for 40 min. After addition of 10 μL of Kinase Detection Reagent (Promega, V6930), plates were incubated for another 30 min at room temperature. Luminescence was measured with a plate-reading luminometer (integration time 750 ms/well). Data were analysed using GraphPad Prism version 8.3.0 (GraphPad Software, San Diego, California, USA).

### Flow cytometry analysis

Following shRNA transduction and selection, CEM-T4 HIV GagzipGFP cells depleted of CLK1 were induced for HIV-1 gene expression with Dox only or Dox and prostratin for 24 h. Following induction, 50% of cell suspension was harvested for western blot analysis and remaining cell suspension was processed for flow cytometry. For the analysis, cells were washed three times with ice-cold PBS and stained for viability using LIVE/DEAD™ Fixable Aqua Dead Cell Stain (Thermo Fisher Scientific, Cat# L34966) for 30 min at 4 °C in PBS according to manufacturer’s protocol. Following staining, cells were fixed with 1% PFA in PBS for 20 min at 4 °C and analyzed for expression of GFP using LSR Fortessa X20-UV cytometer (Becton–Dickinson). Data were analyzed using FlowJo version 10 software (Becton–Dickinson).

To examine the effect of different classes of latency-reversing agents (LRAs) upon CLK1 depletion, CEM-HIV* cells transduced with either control or CLK1-targeted shRNAs were induced with Dox only or Dox+/− an LRA for 24 h. LRAs used included the PKC activators prostratin (2.56 µM) and bryostatin (25 nM), the histone deacetylase (HDAC) inhibitor panobinostat (30 nM), and the BET bromodomain inhibitor JQ1 (2 µM). Cells were harvested 24 h after induction for analysis of HIV-1 GagGFP expression.

For flow cytometry analysis of primary cells, macrophage samples were Fc blocked with Human TruStain FcX (BioLegend, Cat#422302) as per the manufacturer’s instructions. Once the Fc blocking was completed, macrophages and CD4^+^ T cells were surface stained with anti-CD14-Pacific Blue (BioLegend, Cat#301828—macrophages only), anti-CD3-Pacific Blue (BioLegend, Cat#300330—CD4+ T cells only), anti-CD4-APC (BioLegend, Cat#317416), and LIVE/DEAD Fixable Blue (ThermoFisher, Cat#L34962), fixed and permeabilized using BD CytoFix/CytoPerm Fixation/Permeabilization Kit (BD Biosciences, Cat#554714), and stained for the HIV Gag protein using anti-Gag p24-FITC (Beckman Coulter, Brea, CA, Cat#6604665). Flow cytometric data were acquired using a FACSCanto instrument with FACSDiva software (BD Biosciences). All data were analyzed using FlowJo 10.6.0 software (FlowJo, LLC, Ashland, OR). Uninfected cell samples were used to draw the infected cell gate. Infected cells were considered LIVE/DEAD^Neg^, CD14^Int/Pos^CD4^−^p24^+^ (macrophages) or CD3^Pos^CD4^−^p24^+^ (CD4+ T cells). Frequencies of this population were compared between DMSO, Lamivudine/Lopinavir + Ritonavir, and 1H3-treated samples. Within the infected population, the Gag mean fluorescence intensity (MFI) was also compared.

### Statistical analysis

In vitro experiments were performed on at least three independent occasions and are represented as mean ± standard errors of the experiment. Comparisons of statistical significance between two samples were calculated using student’s t-test (two-tailed, Microsoft Excel and Graphpad Prism 8.0). Significant differences represent comparisons to either sh control or DMSO-treated control from induced cells and significance of results is indicated on each graph as follows: p value ≤ 0.05, *, p value ≤ 0.01, **, and p value ≤ 0.001, ***, unless otherwise indicated.

## Supplementary Information

**Additional file 1: Table S1.** Structure and anti-HIV activity of related compounds present in PKIS library. **Table S2.** shRNA vectors used. **Table S3.** Primary antibodies used in the study. **Figure S1.** Effect of SR kinase depletion or inhibition on HIV-1 expression in J-Lat 10.6 cells. **a** Schematic of HIV-1 provirus present in J-Lat 10-6 cells. **b** J-Lat 10.6 cells were infected with lentiviruses expressing shRNAs to the SR kinase indicated and transduced cells were selected with puromycin for 72 h. Following selection, prostratin (2.56 µM) was added to induce HIV-1 gene expression and cells harvested after 24 h for western bot analysis of effects on HIV-1 Gag and GFP expression. **c, d** Cells were treated with DMSO, 1H3 (200 nM), or 2E3 (100 nM) and HIV-1 expression induced with prostratin. After 24 h, cells were analyzed for effects on **c** HIV-1 protein levels and **d** RNA accumulation. Data are indicated as mean ± SEM, n = 4 independent experiments, **p ≤ 0.01, and ***p ≤ 0.001. Dotted vertical lines on the blots represent cropping of lanes on the same representative blot to show compound-treated lanes adjacent to DMSO control lanes. **Figure S2.** Effect of SR kinase depletion or inhibition on HIV-1 MS RNA Splicing. CEM-HIV* were either **a** infected with shRNA expressing lentiviruses to deplete indicated SR kinases or **b** treated with CLK inhibitors. 24 h after induction with Dox and prostratin, cells were harvested, RNA isolated, and RT-PCR performed to detect HIV-1 MS RNAs. Shown on the left are representative gels and, on the right, a summary of n > 3 independent samples. **Figure S3.** HIV-1 TAR and R-U5-Gag transcription profiles in CEM-HIV* cells. CEM-HIV* cells were uninduced (mock), induced with Dox, or Dox + prostratin for 24 h and cells harvested 24 h post-induction for RNA analysis by digital RT-qPCR. Measures of TAR or R-U5-Gag RNA were normalized to ß2M and results expressed as copy number per µg total RNA. Data are indicated as mean ± SD. **Figure S4.** Effect of SR kinase depletion or compound treatment on SR protein levels. **a** Depletion of CLK1 or CLK2 differentially affects abundance of select SR proteins. CEM-HIV* cells were infected with shRNA lentivirus against indicated SR kinases and transduced cells selected with puromycin for 72 h. Following selection, Dox + prostratin was added to induce HIV-1 gene expression and cells harvested for western blots. On the left are the representative western blots showing the effect of individual SR kinase knockdown on SR protein levels and on the right is the quantitation of the western blots across three independent experiments. Band intensity was quantified relative to induced shRNA control and normalized to total protein using Bio-Rad ImageLab software. Data are indicated as mean ± SEM, *p ≤ 0.05, **p ≤ 0.01, and ***p ≤ 0.001. Dotted vertical lines on the blots represent cropping of lanes on the same representative blot to show shRNA-target depletion lanes adjacent to shControl lanes. **b** Primary CD4+ T cells obtained from healthy donors were treated with DMSO or 200 nM 1H3 and cells harvested for western analysis after 3 days. On the left are the representative blots showing expression levels of indicated SR proteins and on the right is the quantitation of n = 3 blots from three independent donor samples. Band intensity was quantified relative to DMSO control and normalized to total protein using Bio-Rad ImageLab software. Data are indicated as mean ± SEM, *p ≤ 0.05. Dotted vertical lines on the blots represent cropping of lanes on the same representative blot to show 1H3-treated lanes adjacent to DMSO-treated lanes. **Figure S5.** Activation of primary CD4+ T cells changes the expression levels of select SR proteins with different kinetics. (refer Fig. 5). Representative western blots showing the expression of multiple different SR proteins in untreated versus treated/activated CD4+ T cell lysates at 24 h and 48 h post-activation. On the bottom is the quantitation of the blots across at least 3 donors. Band intensity was quantified relative to untreated control and normalized to total protein load using Bio-Rad ImageLab software. **Figure S6.** Characterization of 1H3, 2E3, and 1C8 as inhibitors of CMGC kinases (**a**) Nanosyn in vitro kinase profile of the effect of compounds listed in Table S3 on purified kinases [53]. Results are derived from assays with 196 kinases and only results from the subset whose activity was reduced are shown. Blue color indicates < 10% inhibition, yellow indicates > 70% inhibition. **b** Purified CLK1 was incubated with increasing concentrations of 1C8 and assayed for effect on CLK1 autophosphorylation. **Figure S7.** Alignment of CLK1-3. Shown is an alignment of human CLK1-3, indicating the high degree of conservation in the kinase C-terminal kinase domain and the variation in the N-terminal arginine-serine rich domain.

## Data Availability

Data and materials are available upon request.
