# Supplementary material for: Opposing roles of CLK SR kinases in controlling HIV-1 gene expression and latency
Source: Retrovirology. 2022 Aug 19;19:18. doi: 10.1186/s12977-022-00605-4 (PMC9389714; doi:10.1186/s12977-022-00605-4)
Supplement: Supplementary file 1 — Additional file 1: Table S1. Structure and anti-HIV activity of related compounds present in PKIS library. Table S2. shRNA vectors used. Table S3. Primary antibodies used in the study. Figure S1. Effect of SR kinase depletion or inhibition on HIV-1 expression in J-Lat 10.6 cells. a Schematic of HIV-1 provirus present in J-Lat 10-6 cells. b J-Lat 10.6 cells were infected with lentiviruses expressing shRNAs to the SR kinase indicated and transduced cells were selected with puromycin for 72 h. Following selection, prostratin (2.56 µM) was added to induce HIV-1 gene expression and cells harvested after 24 h for western bot analysis of effects on HIV-1 Gag and GFP expression. c, d Cells were treated with DMSO, 1H3 (200 nM), or 2E3 (100 nM) and HIV-1 expression induced with prostratin. After 24 h, cells were analyzed for effects on c HIV-1 protein levels and d RNA accumulation. Data are indicated as mean ± SEM, n = 4 independent experiments, **p ≤ 0.01, and ***p ≤ 0.001. Dotted vertical lines on the blots represent cropping of lanes on the same representative blot to show compound-treated lanes adjacent to DMSO control lanes. Figure S2. Effect of SR kinase depletion or inhibition on HIV-1 MS RNA Splicing. CEM-HIV* were either a infected with shRNA expressing lentiviruses to deplete indicated SR kinases or b treated with CLK inhibitors. 24 h after induction with Dox and prostratin, cells were harvested, RNA isolated, and RT-PCR performed to detect HIV-1 MS RNAs. Shown on the left are representative gels and, on the right, a summary of n > 3 independent samples. Figure S3. HIV-1 TAR and R-U5-Gag transcription profiles in CEM-HIV* cells. CEM-HIV* cells were uninduced (mock), induced with Dox, or Dox + prostratin for 24 h and cells harvested 24 h post-induction for RNA analysis by digital RT-qPCR. Measures of TAR or R-U5-Gag RNA were normalized to ß2M and results expressed as copy number per µg total RNA. Data are indicated as mean ± SD. Figure S4. Effect of SR kinase depleti [file 12977_2022_605_MOESM1_ESM.docx]

**8. Additional Tables and Figures.**

**Table S1: Structure and anti-HIV activity of related compounds present in PKIS library**

| **Compound/Lab Designation** | **Structure** | **EC_50_**  **(nM)** | **CC_50_ (nM)** |
| --- | --- | --- | --- |
| GW806290X/2E3 | 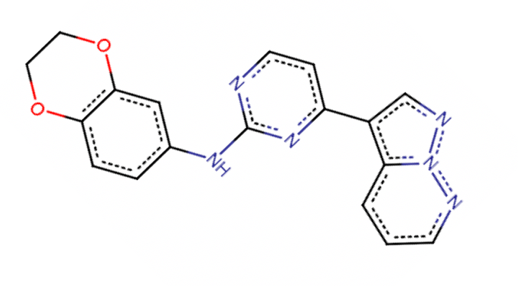 | 5 | 250 |
| GW801372X/1H3 | 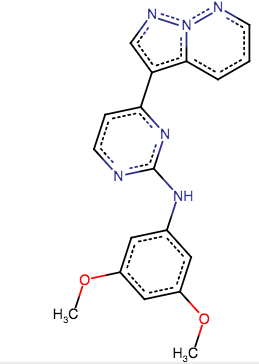 | 10 | 450 |
| GW778894X | 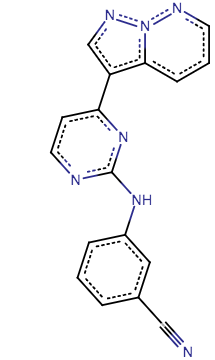 | 15 | ~1000 |
| \| GW779439X \| \| --- \| | 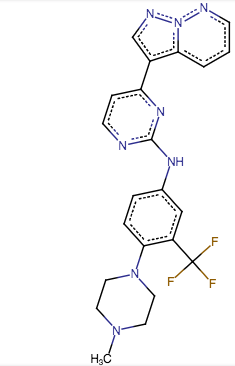 | 15 | ~1000 |
| \| GW780056X \| \| --- \| | 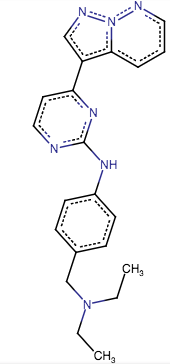 | 15 | ~1000 |
| \| GW781673X \| \| --- \| \|  \| | 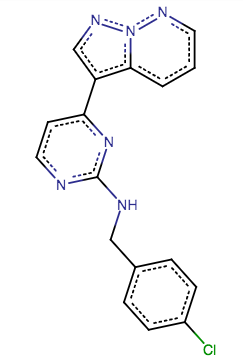 | 16 | >1000 |
| \| GW805758X \| \| --- \| | 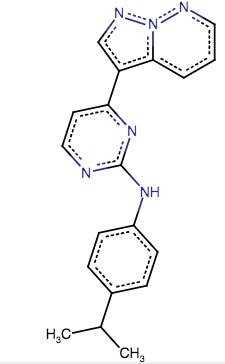 | 62.5 | >1000 |
| GW810372X | 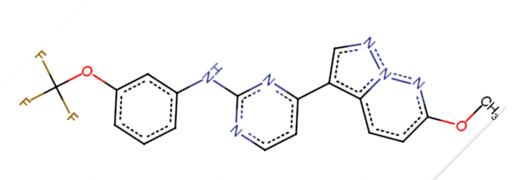 | >1000 | ND |
| GW810372X | 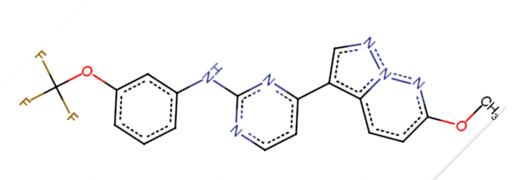 | >1000 | ND |
| GW811761X | 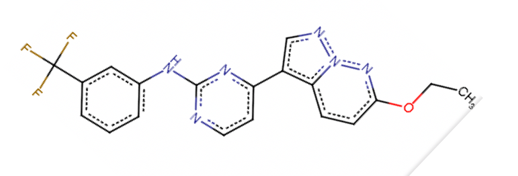 | >1000 | ND |
| GW819077X | 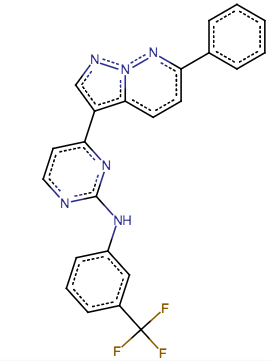 | >1000 | ND |
| GW708336X | 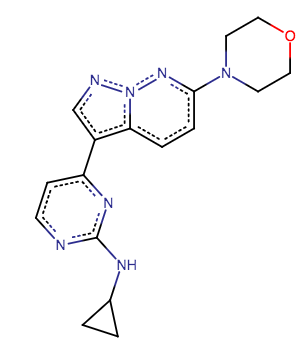 | >1000 | ND |
| \| GW807982X \| \| --- \| | 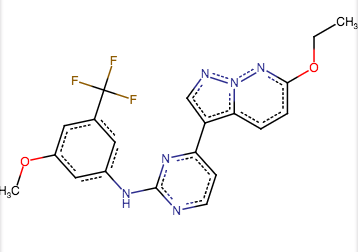 | >1000 | ND |

HeLa C7 cells were incubated with compounds at increasing compound concentration and HIV-1 gene expression induced with Dox (4.5 µM) for 24 h. Cells grown with or without Dox, 1% DMSO served as positive and negative controls, respectively. Dose response on HIV-1 gene expression was measured relative to intracellular GagGFP levels in DMSO & Dox-treated samples. Effects of compounds on cell viability were assessed using alamarBlue assay.

**Table S2: shRNA vectors used**

| **SR kinase** | **shRNA designation** | **Target sequence** | **TRC Clone Name** |
| --- | --- | --- | --- |
| CLK1 |  | TGGTTCATTAAGTACATAGCT | NM_004071.x-1746s1c1 |
| CLK2 |  | CTATCGGCATTCCTATGAATA | NM_003993.2-572s1c1 |
| CLK3 | 1 | CCTTAGATTTCTGCATGAGAA | NM_003992.1-866s1c1 |
|  | 2 | CCTTTGGAGAGGACTACTATG | NM_003992.1-289s1c1 |
| SRPK1 | 1 | GTGGCAATGAAAGTAGTTAAA | NM_003137.x-325s1c1 |
|  | 2 | GAACAACACATTAGCCAACTT | NM_003137.x-1324s1c1 |

**Table S3: Primary antibodies used in the study**

| **Antibody** | **Type** | **Size (kDa)** | **Dilution** | **Company** | **Catalog No.** |
| --- | --- | --- | --- | --- | --- |
| CLK1 | Ms | 60 | 1:1000 in 5% BSA-TBST | Santa Cruz | #515897 |
| CLK2 | Rb | 60 | 1:1000 in 5% BSA-TBST | Abcam | ab65082 |
| CLK3 | Rb | 60 | 1:1000 in 5% BSA-TBST | Abnova | H00001198-M05 |
| SRPK1 | Rb | 110 | 1:2000 in 5% BSA-TBST | Cedarlane | OAAN01583 |
| Gag p24 | Ms | 55, 41, 24 | 1:500 in 1X TBST | NIH AIDS Reagent | #1513 |
| Env gp41 | Hu | 160 | 1:1000 in 1X TBST | NIH AIDS Reagent | #531 |
| Tat 1D9 | Ms | 16, 14 | 1:300 in 1X TBST | NIH AIDS Reagent | #7383 |
| SRSF1 (ASF/SF2) | Ms | 32 | 1:1000 in 1X TBST | Life Technologies | 32-4500 |
| SC35 | Ms | For IF | 1:100 in 5% BSA-PBS | Sigma-Aldrich | S4045 |
| SRSF2 (SC35) | Rb | ~35 | 1:1000 in 1X TBST | Abcam | ab11826 |
| SRSF3 (SRp20) | Ms | 20 | 1:1000 in 1X TBST | Life Technologies | 33-4200 |
| SRSF4 (SRp75) | Rb | 75 | 1:2000 in 1X TBST | Novus Biologicals | NBP2-04144 |
| SRSF5 | Rb | ~40 | 1:1000 in 1X TBST | MBL | RN082PW |
| SRSF6 (SRp55) | Rb | 55 | 1:2000 in 1X TBST | Novus Biologicals | NBP2-04142 |
| SRSF7 (9G8) | Rb | ~35 | 1:2000 in 1X TBST | Abcam | ab137247 |
| SRSF9 (SRp30c) | Rb | 27 | 1:1000 in 1X TBST | MBL | RN081PW |
| SRSF10 (FUSIP1) | Ms | 37 | 1:1000 in 1X TBST | Novus Biologicals | H00010772-M07 |
| Tra2ß | Rb | ~40 | 1:1000 in 1X TBST | Abcam | ab31353 |
| GAPDH | Rb | 35.8 | 1:3000 in 1X TBST | Sigma-Aldrich | G9545 |


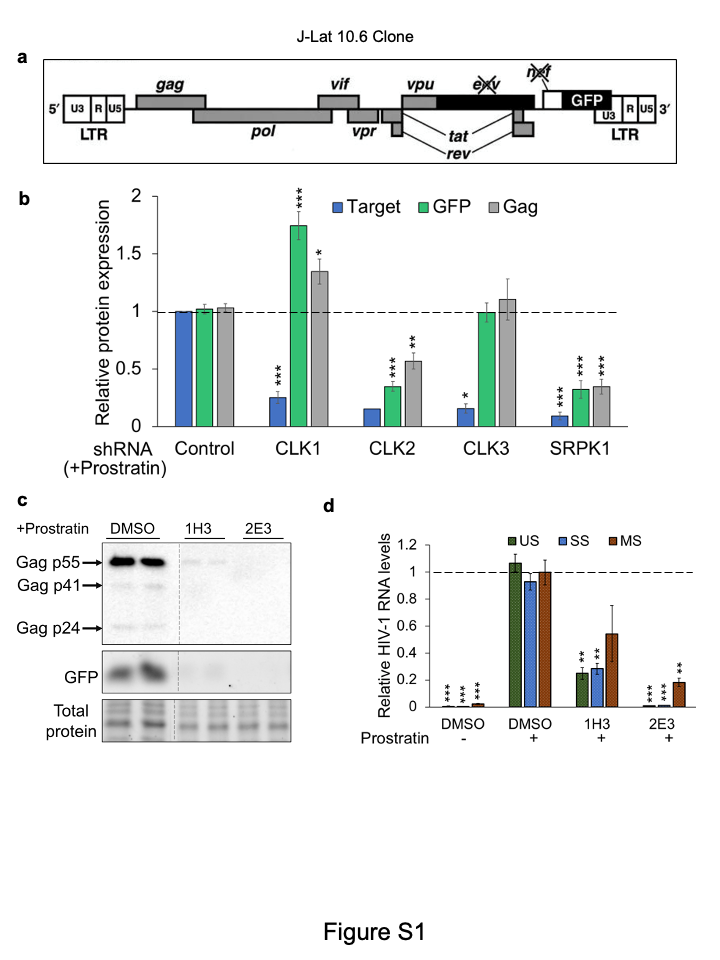


**FIGURE S1. Effect of SR kinase depletion or inhibition on HIV-1 expression in J-Lat 10.6 cells. (a)** Schematic of HIV-1 provirus present in J-Lat 10-6 cells**. (b)** J-Lat 10.6 cells were infected with lentiviruses expressing shRNAs to the SR kinase indicated and transduced cells were selected with puromycin for 72 h. Following selection, prostratin (2.56 µM) was added to induce HIV-1 gene expression and cells harvested after 24 h for western bot analysis of effects on HIV-1 Gag and GFP expression. **(c, d)** Cells were treated with DMSO, 1H3 (200 nM), or 2E3 (100 nM) and HIV-1 expression induced with prostratin. After 24 h, cells were analyzed for effects on **(c)** HIV-1 protein levels and **(d)** RNA accumulation. Data are indicated as mean ± SEM, n=4 independent experiments, **p ≤ 0.01, and ***p ≤ 0.001. Dotted vertical lines on the blots represent cropping of lanes on the same representative blot to show compound-treated lanes adjacent to DMSO control lanes.

**
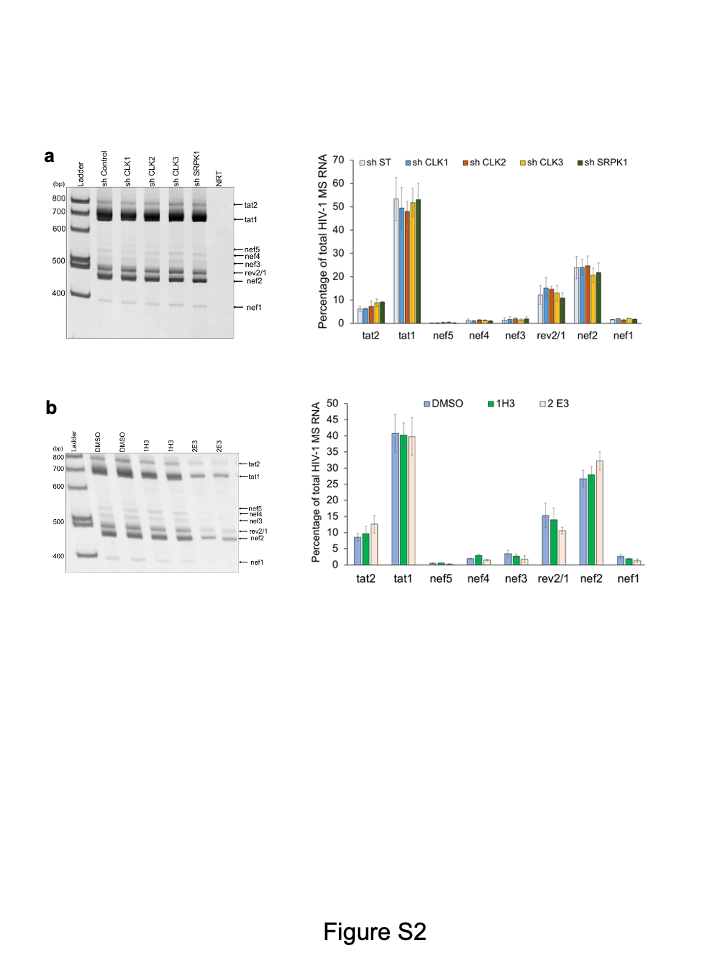
**

**FIGURE S2. Effect of SR kinase depletion or inhibition on HIV-1 MS RNA Splicing.** CEM-HIV* were either **(a)** infected with shRNA expressing lentiviruses to deplete indicated SR kinases or **(b)** treated with CLK inhibitors. 24 h after induction with Dox and prostratin, cells were harvested, RNA isolated, and RT-PCR performed to detect HIV-1 MS RNAs. Shown on the left are representative gels and, on the right, a summary of n>3 independent samples.

**
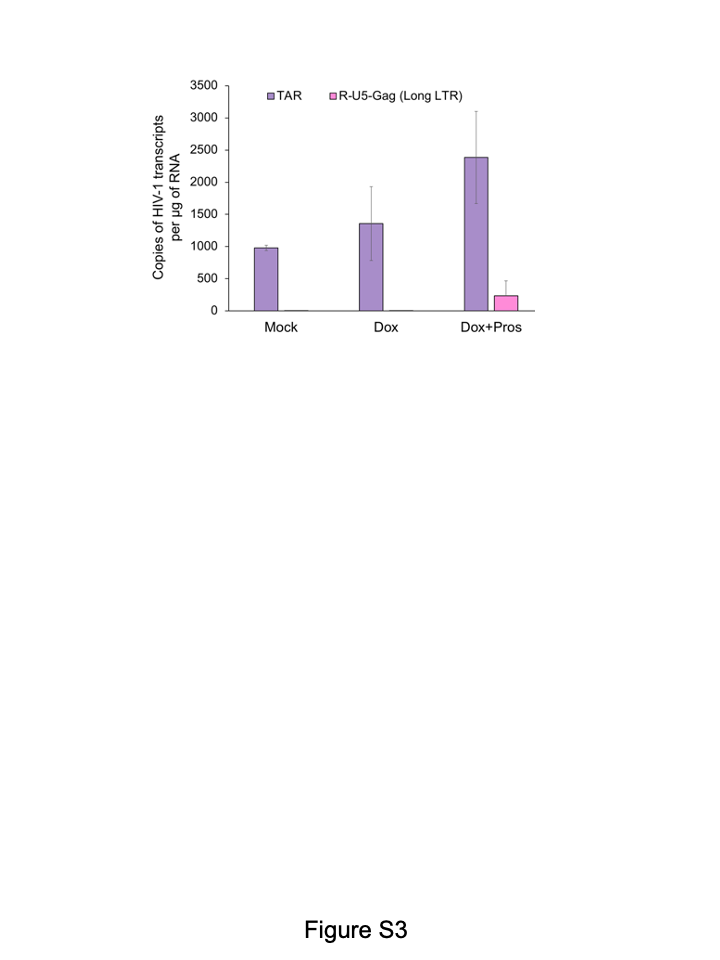
**

**FIGURE S3. HIV-1 TAR and R-U5-Gag transcription profiles in CEM-HIV* cells.** CEM-HIV* cells were uninduced (mock), induced with Dox, or Dox+prostratin for 24 h and cells harvested 24 h post-induction for RNA analysis by digital RT-qPCR. Measures of TAR or R-U5-Gag RNA were normalized to ß2M and results expressed as copy number per µg total RNA. Data are indicated as mean ± SD.

**
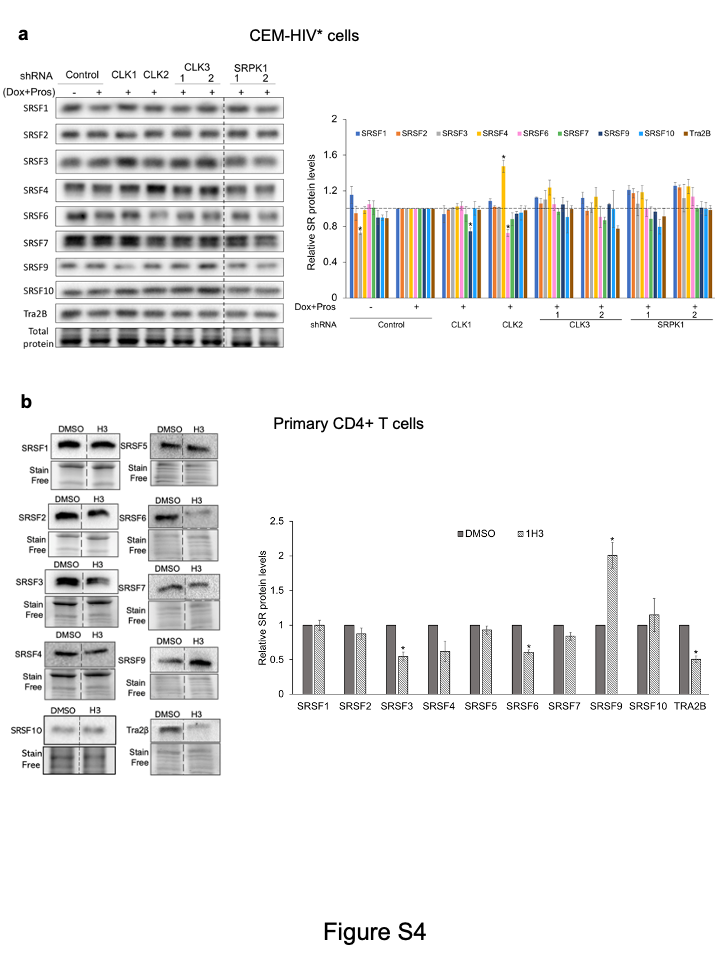
**

**FIGURE S4. Effect of SR kinase depletion or compound treatment on SR protein levels**

**(a)** Depletion of CLK1 or CLK2 differentially affects abundance of select SR proteins. CEM-HIV* cells were infected with shRNA lentivirus against indicated SR kinases and transduced cells selected with puromycin for 72 h. Following selection, Dox+ prostratin was added to induce HIV-1 gene expression and cells harvested for western blots. On the left are the representative western blots showing the effect of individual SR kinase knockdown on SR protein levels and on the right is the quantitation of the western blots across three independent experiments. Band intensity was quantified relative to induced shRNA control and normalized to total protein using Bio-Rad ImageLab software. Data are indicated as mean ± SEM, *p ≤ 0.05, **p ≤ 0.01, and ***p ≤ 0.001. Dotted vertical lines on the blots represent cropping of lanes on the same representative blot to show shRNA-target depletion lanes adjacent to shControl lanes. **(b)** Primary CD4+ T cells obtained from healthy donors were treated with DMSO or 200 nM 1H3 and cells harvested for western analysis after 3 days. On the left are the representative blots showing expression levels of indicated SR proteins and on the right is the quantitation of n=3 blots from three independent donor samples. Band intensity was quantified relative to DMSO control and normalized to total protein using Bio-Rad ImageLab software. Data are indicated as mean ± SEM, *p ≤ 0.05. Dotted vertical lines on the blots represent cropping of lanes on the same representative blot to show 1H3-treated lanes adjacent to DMSO-treated lanes.

**
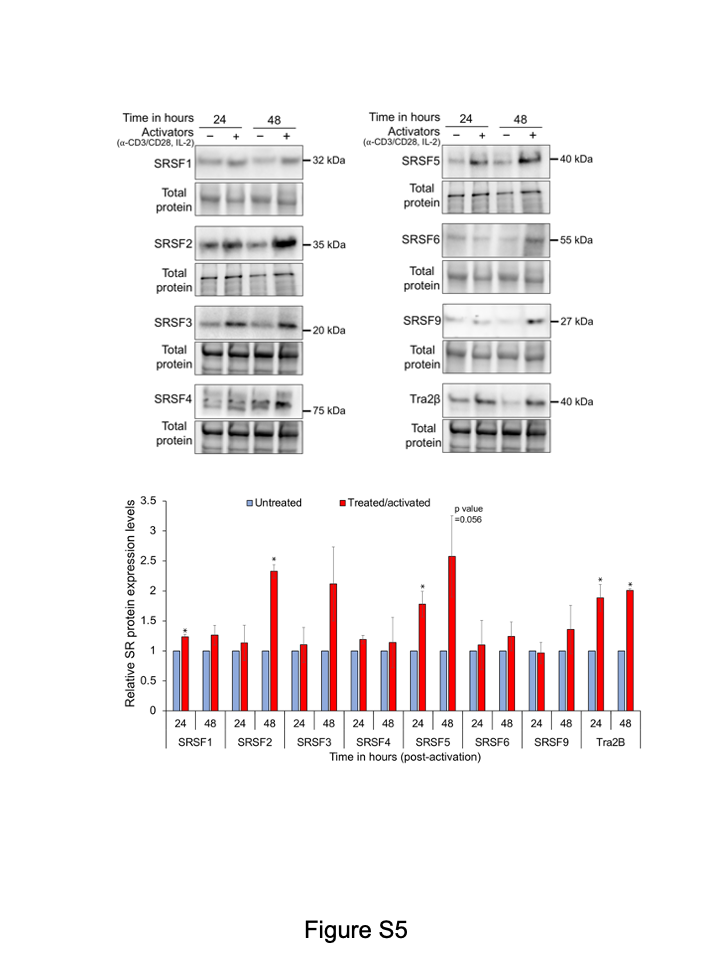
**

**FIGURE S5. Activation of primary CD4+ T cells changes the expression levels of select SR proteins with different kinetics.** (refer Figure 5). Representative western blots showing the expression of multiple different SR proteins in untreated versus treated/activated CD4+ T cell lysates at 24 h and 48 h post-activation. On the bottom is the quantitation of the blots across at least 3 donors. Band intensity was quantified relative to untreated control and normalized to total protein load using Bio-Rad ImageLab software.

**
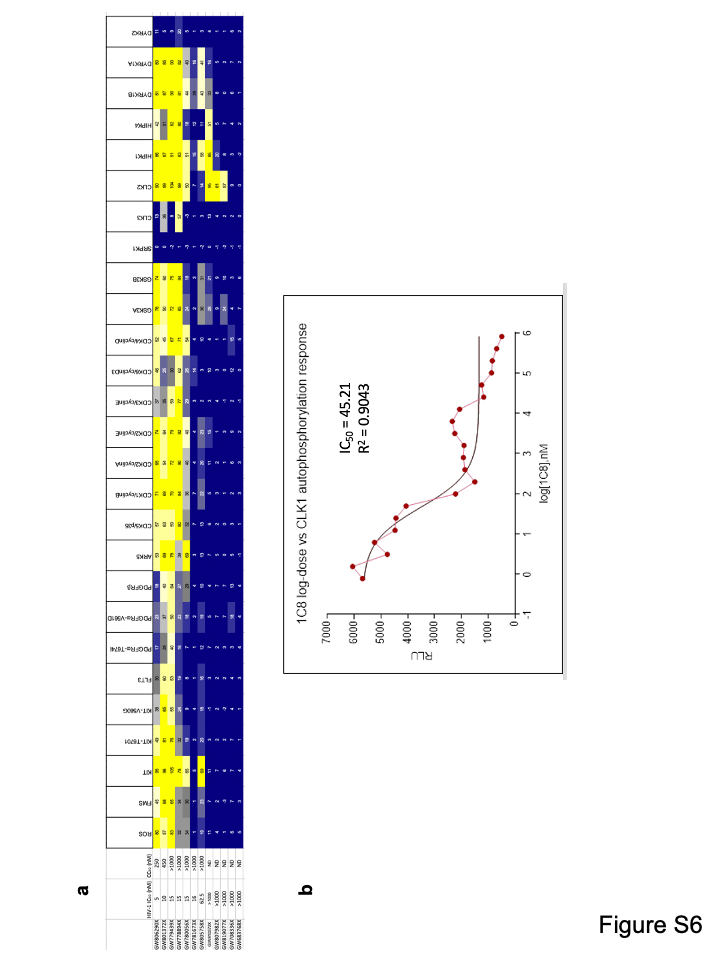
**

**FIGURE S6. Characterization of 1H3, 2E3, and 1C8 as inhibitors of CMGC kinases (a)** Nanosyn *in vitro* kinase profile of the effect of compounds listed in Table S3 on purified kinases [53]. Results are derived from assays with 196 kinases and only results from the subset whose activity was reduced are shown. Blue color indicates <10% inhibition, yellow indicates > 70% inhibition. **(b)** Purified CLK1 was incubated with increasing concentrations of 1C8 and assayed for effect on CLK1 autophosphorylation.

**
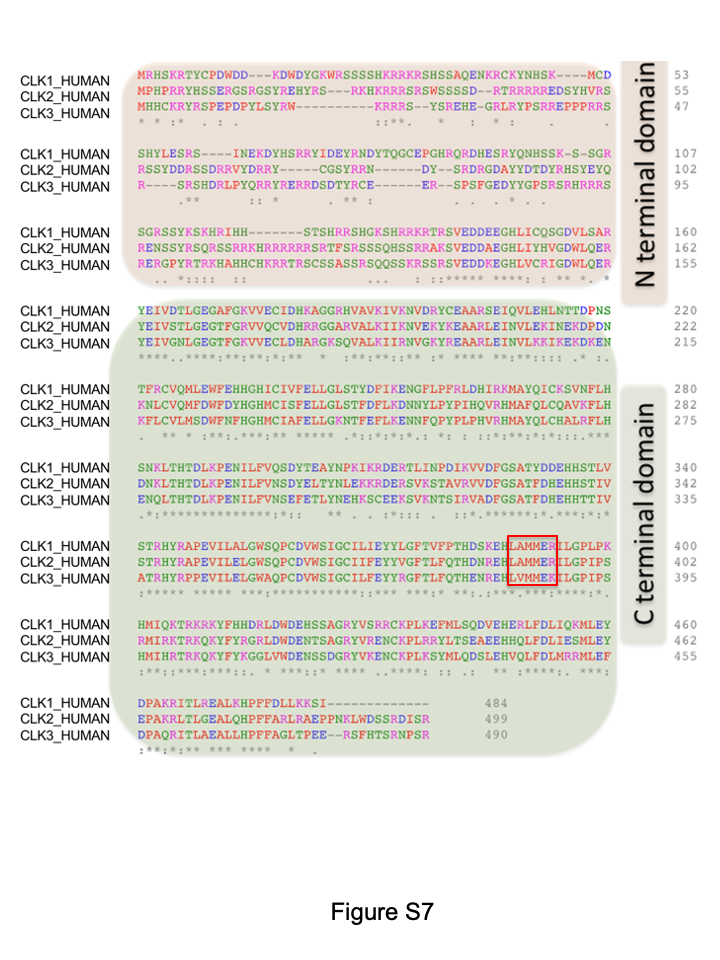
**

**FIGURE S7. Alignment of CLK1-3.** Shown is an alignment of human CLK1-3, indicating the high degree of conservation in the kinase C-terminal kinase domain and the variation in the N-terminal arginine-serine rich domain.
